# Oxidative polymerization of zinc porphyrin using the electrochemical Langmuir–Blodgett trough†

**DOI:** 10.1039/d6ra05196k

**Published:** 2026-08-18

**Authors:** Katarina Majerová Varga, František Vavrek, Veronika Urbanová, Jan Plutnar, Zdeňek Bastl, Markéta Pazderková, Lucie Ludvíková, Paul I. Dron, Magdaléna Hromadová, Lubomír Pospíšil

**Affiliations:** a Institute of Organic Chemistry and Biochemistry Czech Academy of Sciences Flemingovo nám. 542/2 160 00 Prague Czech Republic; b J. Heyrovský Institute of Physical Chemistry, Czech Academy of Sciences Dolejškova 2155/3 182 23 Prague 8 Czech Republic; c Department of Chemistry, Middle East Technical University Ankara 06800 Turkey

## Abstract

Multielectron electrochemical oxidation of non-substituted zinc porphyrin yields a polymeric structure. We report the oxidative formation of polymerization nuclei using the electrochemical Langmuir–Blodgett (LB) trough and an STM-type electrode tip. The electrochemical method eliminates the need for an excess of a chemical oxidant. The electrochemical LB trough assembly, which we reported earlier, efficiently produces a large number of separated thin oxidized nuclei. Topographic profiles from STM images indicate their average height of 0.52 ± 0.04 nm. The most frequently observed islands have an average length of 11.6 ± 0.6 nm. Their growth follows the Volmer–Weber “island growth” model, yielding isolated islands of a new phase. These experiments confirm electrochemical oxidation as an alternative method for synthesizing thin porphene oligomers without an external oxidizing agent.

## Abstract 2

Multielectron electrochemical oxidation of non-substituted zinc porphyrin yields a polymeric structure.

## Graphical abstract

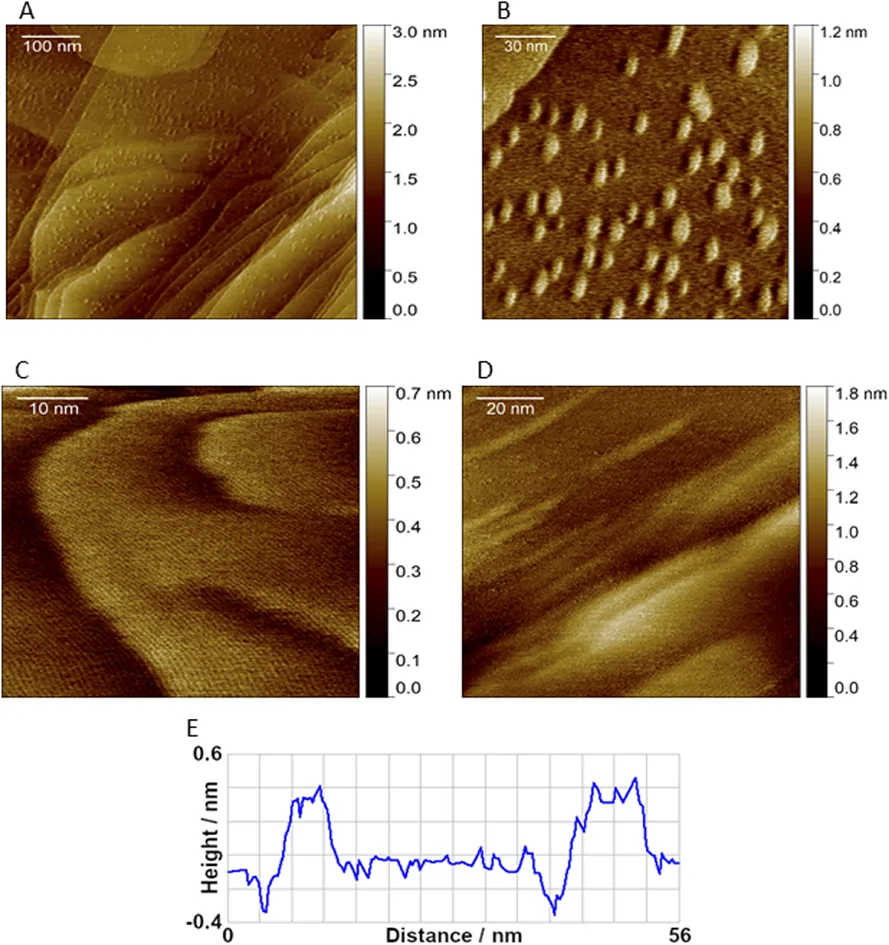

## 1. Introduction

Recently we described the design and testing of the Langmuir–Blodgett (LB) trough enabling the electrochemical measurements in an adsorbed organic layer floating on a water phase.^1^ The motivation was the prospective oxidative formation of low dimensional covalent organic frameworks.

The aim of this communication is to oxidize Zn porphyrin to porphene oligomers in the LB trough using cyclic voltammetry. Langmuir–Blodgett trough arrangement differs from standard voltammetric measurements, which utilize a large area working electrode immersed in the solution containing both Zn porphyrin and supporting electrolyte. In this work, Zn porphyrin (insoluble in the aqueous phase) floats on the surface of the aqueous phase creating a two-dimensional (2D) surface layer. The oxidation starts electrochemically using a small STM-type electrode tip and the polymerization proceeds in the absence of any chemical oxidant in the aqueous phase.

The formation of 2D structures has been widely explored with envisioning of new type of catalysts, solar energy conversion technologies and numerous other applications in materials science.^2–11^ Covalent organic frameworks find application in sensing,^12^ optoelectronic^13^ or separation techniques.^14^ Electrochemistry represents a convenient method for preparation of such materials.^15–17^ Redox-active adsorbed layers at electrodes (Hg, Au, Pt and others) are known for many decades. The first report appeared in 1942 by Brdička^18^ and the theory for voltammetric characteristics was published by Laviron.^19^ Regarding porphyrin-based 2D materials, Vorotyntsev *et al.* used electrochemical oxidative approach to produce 2D polymeric structure by direct linking of the unsubstituted magnesium porphyrin units.^20^ Bulk electrolysis of magnesium porphyrin was used for polymer modification,^21^ whereas the structure of the polymer depended on the applied oxidation potential.^22^ A review of 2D porphyrin arrays with emphasis on phenol- and quinone-substituted derivatives including phase transitions and ordered 2D phase boundaries was published by Ariga *et al.*^23^ Previous works^20–22^ used bulk oxidation of the porphyrin solution on much larger electrode areas, whereas this communication explores polymerization process using ultramicroelectrode (UME) anode in a 2D surface layer. Hence, the polymer yield is expected to be low and probably difficult to analyze. This could be the reason why the electrochemistry on Langmuir–Blodgett films is rarely used.^24^ This communication is an example of using the modified electrochemical LB setup reported previously^1^ (see Fig. S1) for the oxidation of zinc porphyrin proceeding exclusively in the surface layer.

**Chart 1:**
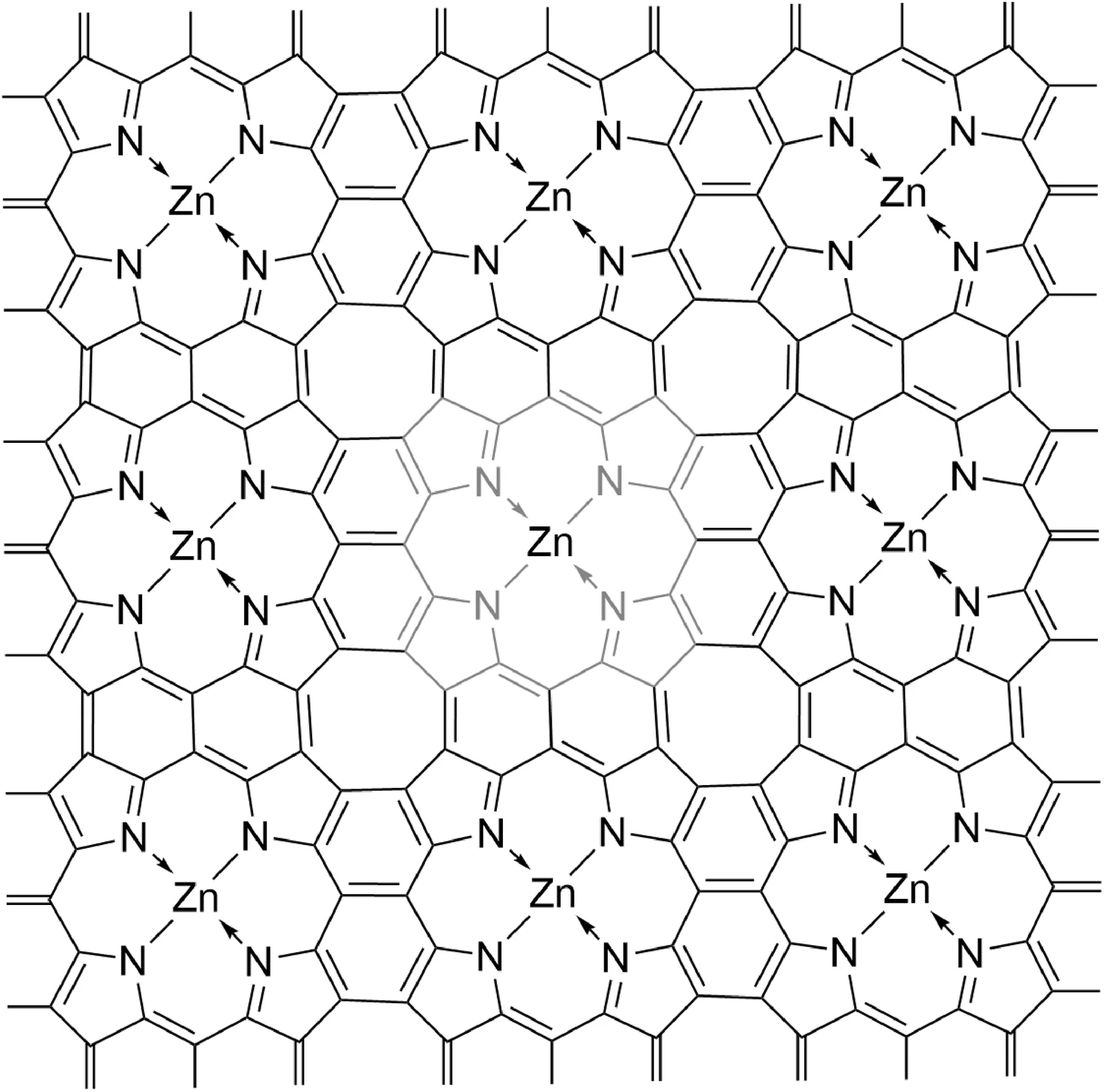
Polymer (Zn porphene) formed by chemical oxidation of Zn porphyrin.

**Scheme 1:**
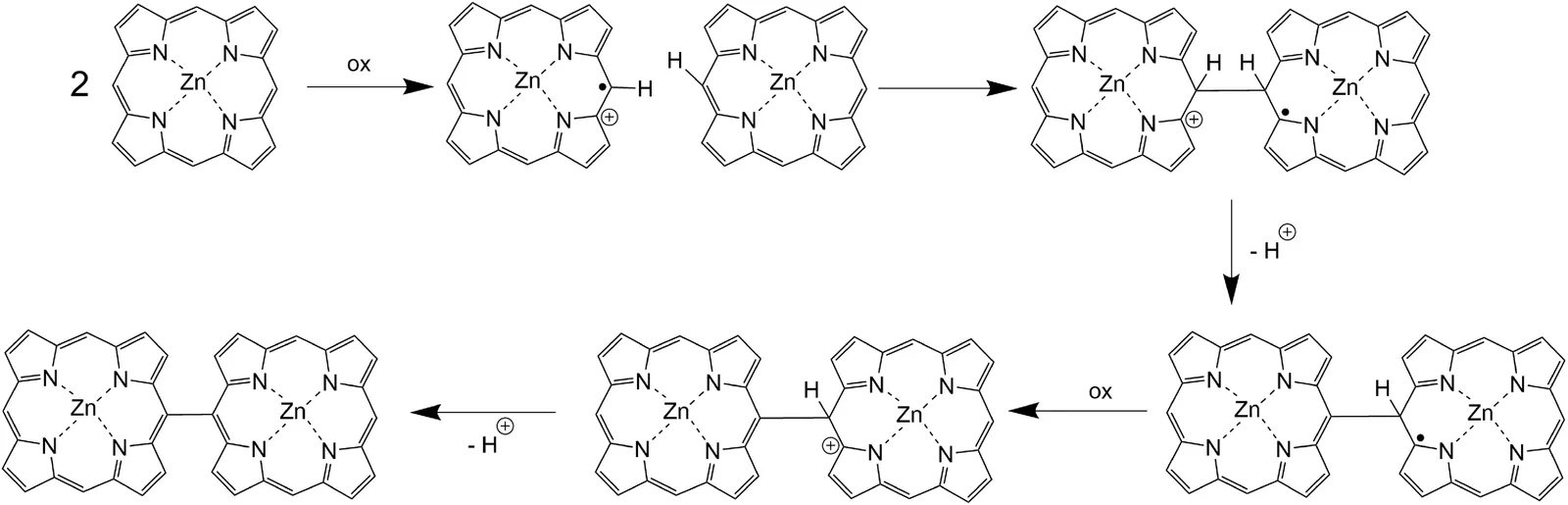
Suggested growth mechanism on the periphery of a nucleus during the film formation.

Chemical oxidation performed by Michl *et al.*^25,26^ reported porphyrin-based polymer similar to graphene (Chart 1). Authors suggested that radical species of the first oxidation step initiate the growth of a nucleus on its periphery resulting in a 2D polymer formation (Scheme 1). Chart 1 shows that this polymer preserves the bonding sites in the porphyrin rings. Sites are available for metalation without losing the hyper-conjugation of the entire system. The properties of porphene sheets and multilayers could be finely tuned. The chemical oxidation^26^ of a bilayer of Zn porphyrin floating on the water surface (chemical oxidant being dissolved in the aqueous phase) produces radical cations (Scheme 1). Electron transfer (ET) occurs at random surface locations (instantaneous nucleation) and each of these growth seeds may produce a sheet of porphene consisting of numerous small single crystalline domains with mutually unrelated lattice orientations.

For growth of a single domain, one should start with a single nucleation site, which is difficult to achieve by chemical oxidation. This contribution attempts to minimize the number of nuclei (ideally to one) formed during an electrochemical anodic oxidation using the STM-type UME, which may serve this purpose. The seed growth speed should depend on the carefully selected anodic potential. The oxidation of the initial monomer requires a higher anodic potential than the oxidation of the dimer.^27^ A small number of growth seeds move away from the tip electrode through electric repulsion of cations by a positive electrode charge, interaction with counter ions or solvent solubility. The use of a point anode centered in a circular cathode, both touching the surface can ensure uniform lines of force concentrated at the surface. However, the growth of domains on top of each other^8^ may not be excluded. The probable mechanism of growth may include Volmer–Weber or “island growth”, Frank–van der Merwe growth or “layer-by-layer growth”, Stranski–Krastanov growth or “island on layer”.^28^ The growth type depends on the strength of the adsorbate–adsorbate and adsorbate–electrode interactions. Surface electrostatics, ion pairing and anion–cation salt formation plays also an important role.^29^

The aim of this paper is to use an electrochemical oxidation of Zn porphyrin as an alternative method to chemical oxidation. Here an electrode is a “redox agent”. Promising interfacial electrochemistry method for the formation of thin 2D membrane of Zn porphene without the necessity to use chemical oxidant in the aqueous phase is explored here.

## 2. Experimental section

### 2.1. Materials and methods

Synthesis of Zn porphyrin was performed by authors following the published procedure. Identification of compounds was determined by standard methods (elementary analysis, NMR, IR spectra).^30–32^ Other chemicals, ZnCl_2_, indifferent electrolytes (NaCl, NaClO_4_ and tetra butylammonium hexafluorophosphae) and solvents (acetonitrile, 1,2-dichloroethane, chloroform) were obtained from Sigma-Aldrich. Ultrapure water (ohmic resistivity 18.2 MΩ cm) was obtained from Merck Millipore water purification system. The construction of the Langmuir–Blodgett trough modified for electrochemical measurements (see Fig. S1) was described recently.^1^ The whole piston assembly is placed inside a housing. It is sealed with a radial shaft seal for isolation from the liquid, still allowing vertical motion. The insulation of the UME electrode tip was achieved by clamping the PTFE tubing with a piston rod having a threaded end. The working electrode tip was made from a 90% Pt–10% Ir alloy wire with a diameter of 0.25 mm dipped into an aqueous etching solution (1 M CaCl_2_ and 0.25 M HCl) at one end. The wire was electrochemically AC etched at one end. The shape of the resulting tip was inspected under an optical microscope and a scanning electron microscope (Nova NanoSEM 450, FEI). It revealed a circular cone shape with a pointed apex. The etched wire was then sheathed inside of a PTFE capillary tube (1.5 mm OD and 0.25 mm ID, Metrohm, Czech Republic), allowing the etched tip to protrude ∼2 mm and the other end ∼5 mm, providing sufficient wire length for later supply wire soldering. In the next step, the sheathed etched tip was dipped in molten polyethylene (low-density PE, Sigma-Aldrich). Plasma induced polymer grafting was afterwards used to adjust the contact angle with water to minimize the perturbation of a flat aqueous surface. First, the electrode tip was left in a plasma cleaner at 60 W for 30 s and then it was immediately immersed in 10% aqueous acrylic acid for 1.5 h at room temperature. Finally, the electrode tip was thoroughly washed with ultrapure water. Electrochemical active area of UME was evaluated by cyclic voltammetry using known reversible system of 1 mM potassium ferrocyanide in aqueous 0.1 M Na_2_SO_4_.^33^ A representative cyclic voltammogram is shown in Fig. S2. Measured currents were used for evaluation of the electrode size and quality. Only 20–30% of tips passed such test. The collection of tips had area ranging from 0.2 to 40 µm^2^. Tips with two different surface areas were used, 0.9 µm^2^ and 4.5 µm^2^, respectively. The LB layer contained 0.1 mL of a solution of 0.08 mg mL^−1^ of Zn porphyrin in chloroform and was left undisturbed for 60 min to evaporate the solvent. Electrochemical methods used potentiostat CompacStat.h (Ivium Technologies, The Netherlands) and included slow voltage scan cyclic voltammetry (2–50 mV s^−1^) and long-time chronoamperometry (5000–10 000 s) at various constant potentials. The reference electrode was Ag|AgCl|1 M KCl. The circular counter electrode was in the adsorbed layer (see Fig. S1 and our previous publication^1^).

The measurement procedures followed subsequent steps:

(i) STM-type anode tip is in the aqueous phase.

(ii) Solution of Zn porphyrin in chloroform is spread on the aqueous phase and let evaporate (60 min).

(iii) The resulting adsorbed film is then compressed and measurements at a constant pressure are performed.

(iv) The piston with the anode tip is moved through the aqueous phase up towards the surface layer in nanometer steps (see Fig. S5). Voltammograms are measured after each step up until the first contact with the surface layer is detected (Fig. 2).

(v) The upward movement of the anode continues and stops when the same voltammograms are repeatedly obtained.

(vi) Suitable potentials for attempting the oxidative polymerization are evaluated from those voltammograms.

(vii) Chronoamperometry at one of the constant selected potentials continues for 5000 or up to 10 000 seconds. The current decay is slow, which indicates no chance to achieve complete exhaustion in real time.

Scanning tunneling microscopy (STM) measurements were performed on Agilent 5500 SPM (Agilent Technologies, USA). Samples for STM studies used flame-cleaned monocrystalline 1 cm^2^ Au(111) plates. At the end of chronoamperometry, the Au plate was raised from the water phase through the surface layer (no potential applied), rinsed with ultrapure water and dried. This followed a procedure used previously.^26^

The X-ray photoelectron spectra of the sample were acquired using a modified ESCA 3 MkII multi-technique spectrometer (VG Scientific, East Grinstead, UK) equipped with a hemispherical electron analyzer operated in a fixed transmission mode. The binding energy scale of the spectrometer was calibrated using the Au 4f_7/2_ (84.0 eV) and Cu 2p_3/2_ (932.6 eV) photoemission lines. Binding energy steps for high-resolution scans were 0.1 eV with 200 ms dwell time per step. Measurements were performed at room temperature. The pressure in the XPS analysis chamber during spectra acquisition was 5 × 10^−9^ mbar. The spectra were collected at a takeoff angle of 45° with respect to the macroscopic surface normal. High-resolution C 1s, N 1s, O 1s, and Zn 2p_3/2_ photoelectron spectra were measured. Individual components of the deconvoluted spectra were described by a convolution of a Lorentzian function, representing the lifetime broadening, and a Gaussian function to account for the instrumental resolution. The Gaussian broadening was kept the same for different components. Shirley background was used to account for the inelastic scattering of photoelectrons in the high-resolution spectra. Quantification of the elemental concentrations was accomplished by correcting the photoelectron peak intensities for tabulated cross sections and the analyzer transmission function using CasaXPS software.

Raman spectra were measured on a Renishaw inVia™ Qontor® upright confocal Raman microscope using a 532 nm excitation, 1800 lines per mm grating and a LWD 50× objective (NA 0.55, LWD 8.2 mm, Leica). Spectral processing included cosmic ray removal, baseline correction using a polynomial function and, in case of a Zn porphyrin powder, Savitzky–Golay smoothing (using a 9th order polynomial function).

Brewster angle microscopy (BAM) images were recorded on in-house built microscope (Fig. S3) consisting of 1 ns-pulsed laser (Ekspla NT242) tuned to 532 nm, two Glan-laser calcite polarizers, silver mirrors, OLYMPUS objective 10×, achromatic tube lens (175 mm FL), THORLABS CMOS camera (2448 × 2048 pixels).

## 3. Results and discussion

This communication is an attempt to form a 2D layer of Zn porphene by electrochemical oxidation of Zn porphyrin, which is adsorbed on an aqueous electrolyte|air interface in the LB trough. Zn porphyrin is insoluble in the aqueous phase. STM-type ultramicroelectrode was used as the working electrode to overcome a problem with low electrical conductivity of the Zn porphyrin layer. The environment for electron transfer in the LB layer excludes any electrolyte and solvent usually providing the electronic conductivity between the working electrode and the counter electrode. Such conditions need to be tested by voltammetric measurements in the bulk solutions first.

### 3.1. Electrochemistry of Zn porphyrin in solution

**Fig. 1:**
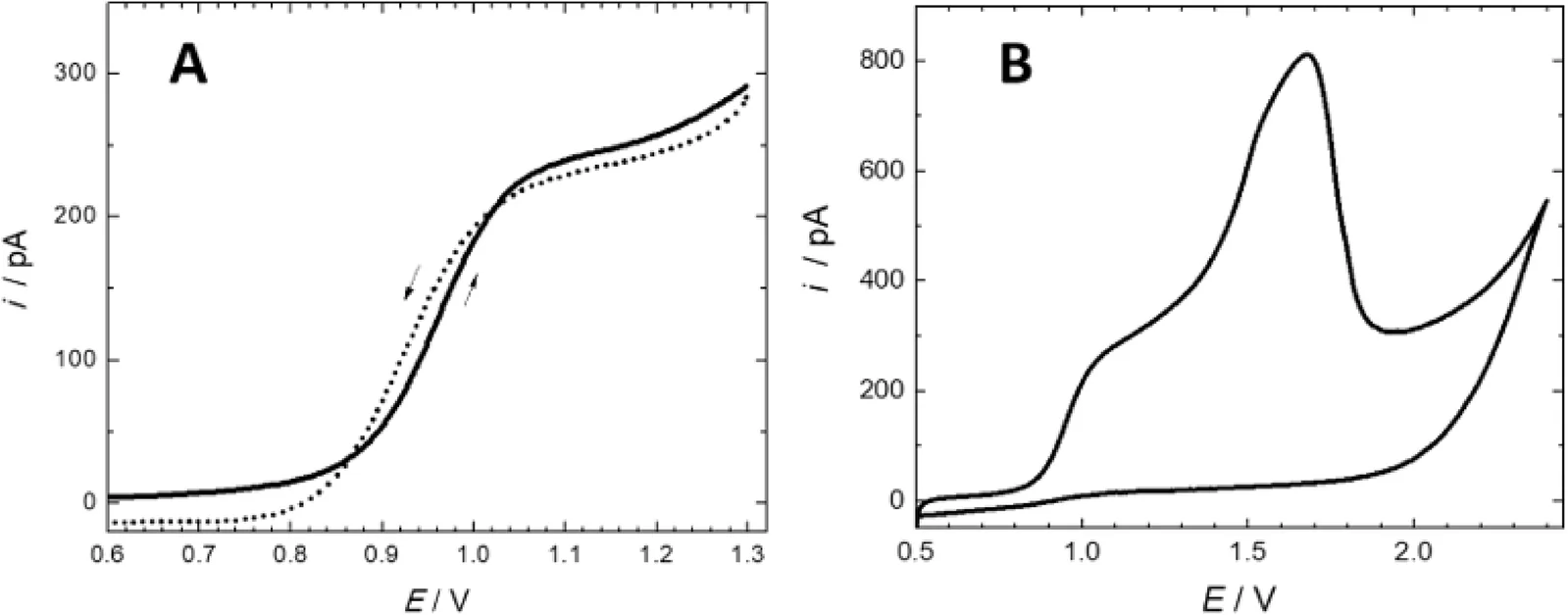
(A) Cyclic voltammogram of 1.4 mM Zn porphyrin in 1,2-dichloroethane without any supporting electrolyte using freshly polished Pt UME of 10 µm diameter and 2 mV s^−1^ scan rate. (B) Cyclic voltammogram to the extended potential range of 2.4 V.

Our first experiments of cyclic voltammetry used Pt UME of 10 µm for Zn porphyrin oxidation in pure 1,2-dichloroethane solvent, see Fig. 1. Representative baseline for 10 µm Pt UME in pure 1,2-dichloroethane is shown in Fig. S11. The first oxidation wave of Zn porphyrin is chemically reversible and is located at 0.96 V (Fig. 1A). The reversibility confirms the absence of any self-inhibition by an oxidation product. A linear log-plot analysis of Zn porphyrin of the first oxidation wave is given in Fig. S4. It shows a finite electron transfer rate with the transfer coefficient *α* = 0.5.

The extended potential range from 0 V up to 2.4 V shows the second oxidation step at 1.5 V, which gives a higher current compared to the first oxidation step (Fig. 1B). The second oxidation process in Fig. 1B is expected to have an S-shape similar to the wave in Fig. 1A. Instead, the current falls down between 1.7 V and 2 V to a current level of the first wave. This decay indicates that the oxidation inhibits the ET by adsorption. The UME needs polishing prior to each measurement. These characteristics of the second oxidation suggest a possible formation of a dimer (or oligomers) responsible for this inhibition (see Scheme 1).^3^

### 3.2. Cyclic voltammetry of Zn porphyrin in LB surface layer

**Fig. 2:**
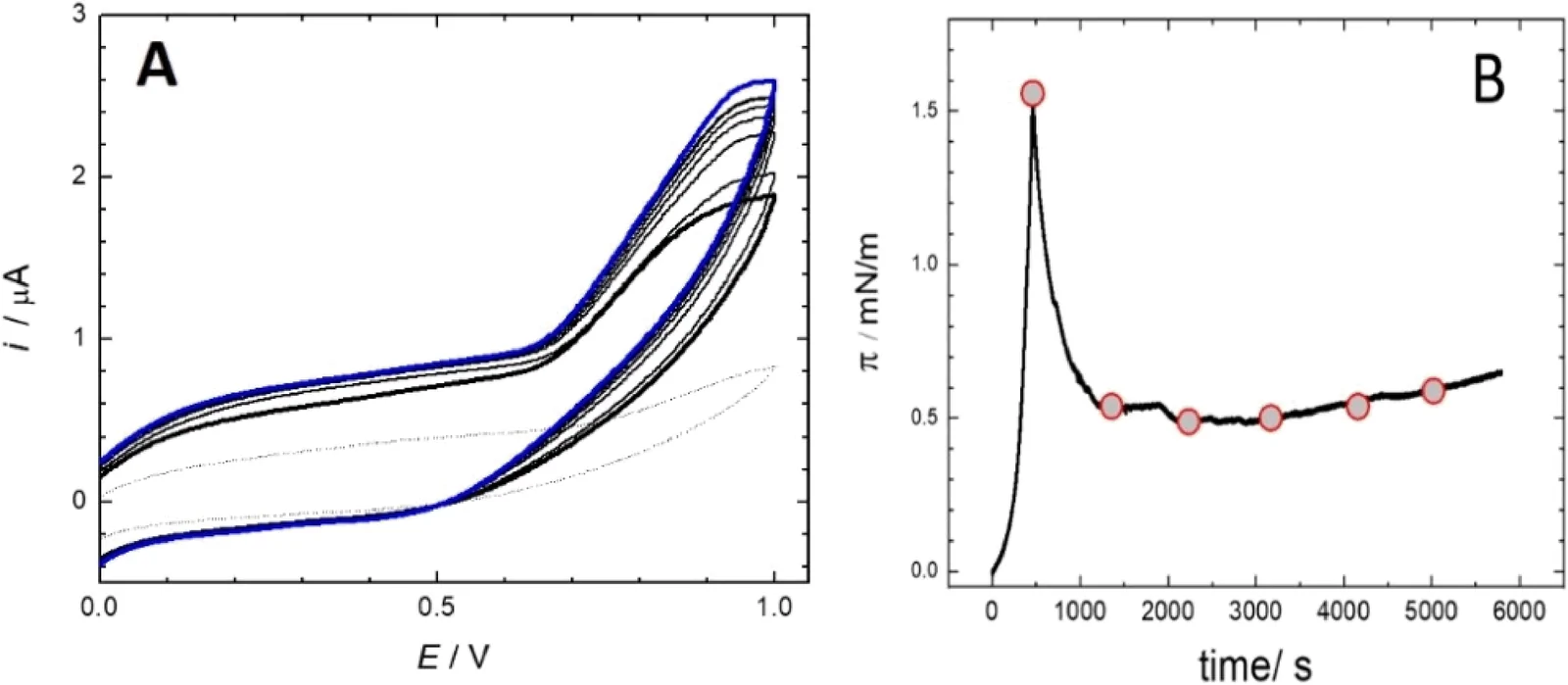
(A) Cyclic voltammograms of the first oxidation wave of Zn porphyrin at different tip positions. Dotted curve corresponds to the tip position in the aqueous phase representing the baseline. Thick black curve corresponds to first contact with LB layer; thick blue curve corresponds to the full immersion of the electrode into the LB layer. Tip surface area was 4.5 µm^2^. (B) Time evolution of the surface pressure in the LB layer of Zn porphyrin on 0.1 M NaCl aqueous phase at 0.1 mm s^−1^ barrier compression rate. The barrier compression rate was 1 mm s^−1^ and movement of the barrier was stopped after 1000 s (B). The movement of the UME tip started at constant pressure as described in Methods. Circles indicate approximate times at which cyclic voltammograms were measured.

The surface layer was prepared by spreading 100 µL of Zn porphyrin solution in chloroform on the aqueous phase. The STM tip position was raised by a micro-screw for rough regulation. Further upward displacement was controlled by a piezoelectric actuator (Fig. S1). Repeated cyclic voltammograms recorded inside the voltage range of the first wave (Fig. 2A) showed a gradual increase of the limiting current (constant current of the S-shaped curves) until it reached a stable maximum value. The change of the maximum peak current with the tip displacement towards the surface is given in Fig. S5. Further tip movement through the layer leads to the loss of the electric contact. The LB time dependence of surface layer pressure is shown in Fig. 2B. The mean molecular area of porphyrins is notoriously poorly reproducible.^26^ We found values in the range from 61 Å^2^ to 72 Å^2^.

**Fig. 3:**
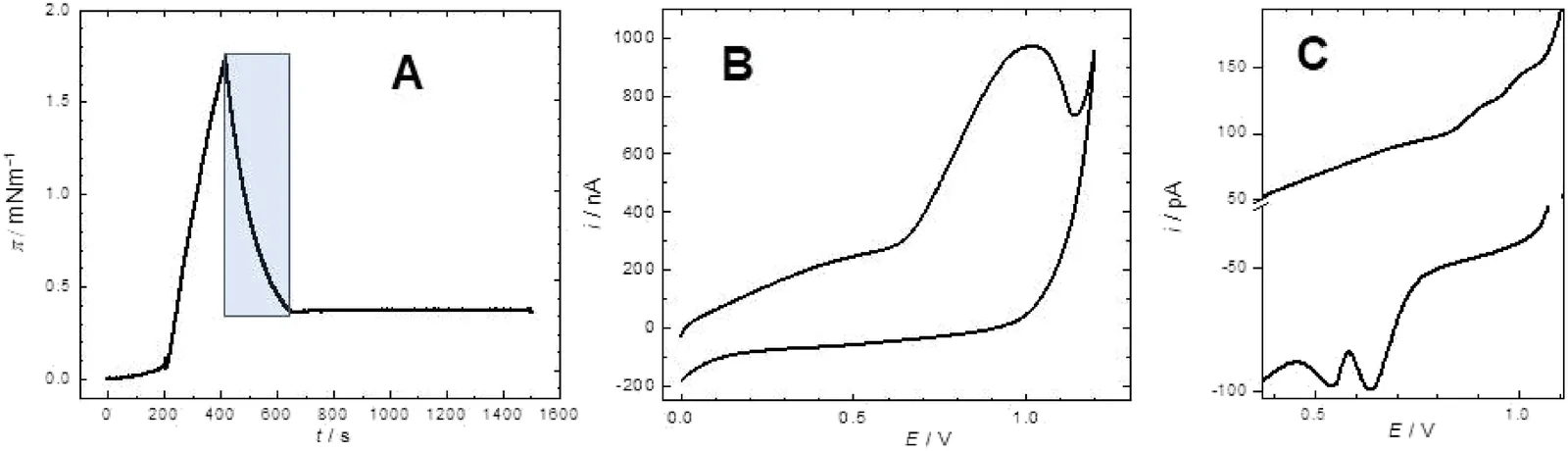
(A) Time evolution of the surface pressure of Zn porphyrin spread on 0.1 M NaCl aqueous phase. Initial decrease of surface pressure is marked by a rectangle. (B) Cyclic voltammogram recorded during the marked time interval in (A), where the pressure decreases by film reorganization (B). The barrier compression rate was 1 mm s^−1^ and movement of the barrier was stopped after >600 s. Subsequent voltammogram at the flat part of the time > 600 s is a zoomed detail of a voltammetric scan up to 1.5 V (C). The aqueous phase was 0.1 M NaCl. The voltage scan rate was 10 mV s^−1^.

The lowering of pressure indicates the change of the film structure during the compression. Electrochemistry measurements in LB film have several specific features, which have to be considered. The very first voltammogram taken during the pressure drop (Fig. 3A) reflects the reorganization of the surface layer accompanied by a change of the interfacial capacity. The capacitive current at 0.4 V decreases from ∼200 nA (Fig. 3B) obtained during the surface pressure decrease to ∼50 pA (Fig. 3C) measured within the flat part of the surface pressure graph (Fig. 3A). For a better comparison, Fig. 3C is a zoomed detail of a voltammogram scanned to 1.5 V and back. All following voltammograms measured with the STM-type tip in the surface layer show a two-step oxidation. Two reduction peaks of the oxidized Zn porphyrin are detected at 0.62 V and 0.53 V. According to our previous investigation of dimer formation,^27^ reduction currents are assigned to a dimer reduction.

The height of the oxidation peak in Fig. 3B is about 900 nA, whereas very small oxidation peaks close to 1 V could correspond to the dimer reoxidation, since the potential scan in Fig. 3C starts from positive voltage. Note that the dimer reduction peak currents are only ∼50 pA in height (Fig. 3C), which makes the reduction/reoxidation comparable. Since the current in Fig. 3B is ∼900 nA, small dimer peaks ∼50 pA do not show up in Fig. 3B. Several potentials (1.35 V, 1.4 V, 1.6 V and other) were tested. No substantial difference in the oxidation pattern was found.

**Fig. 4:**
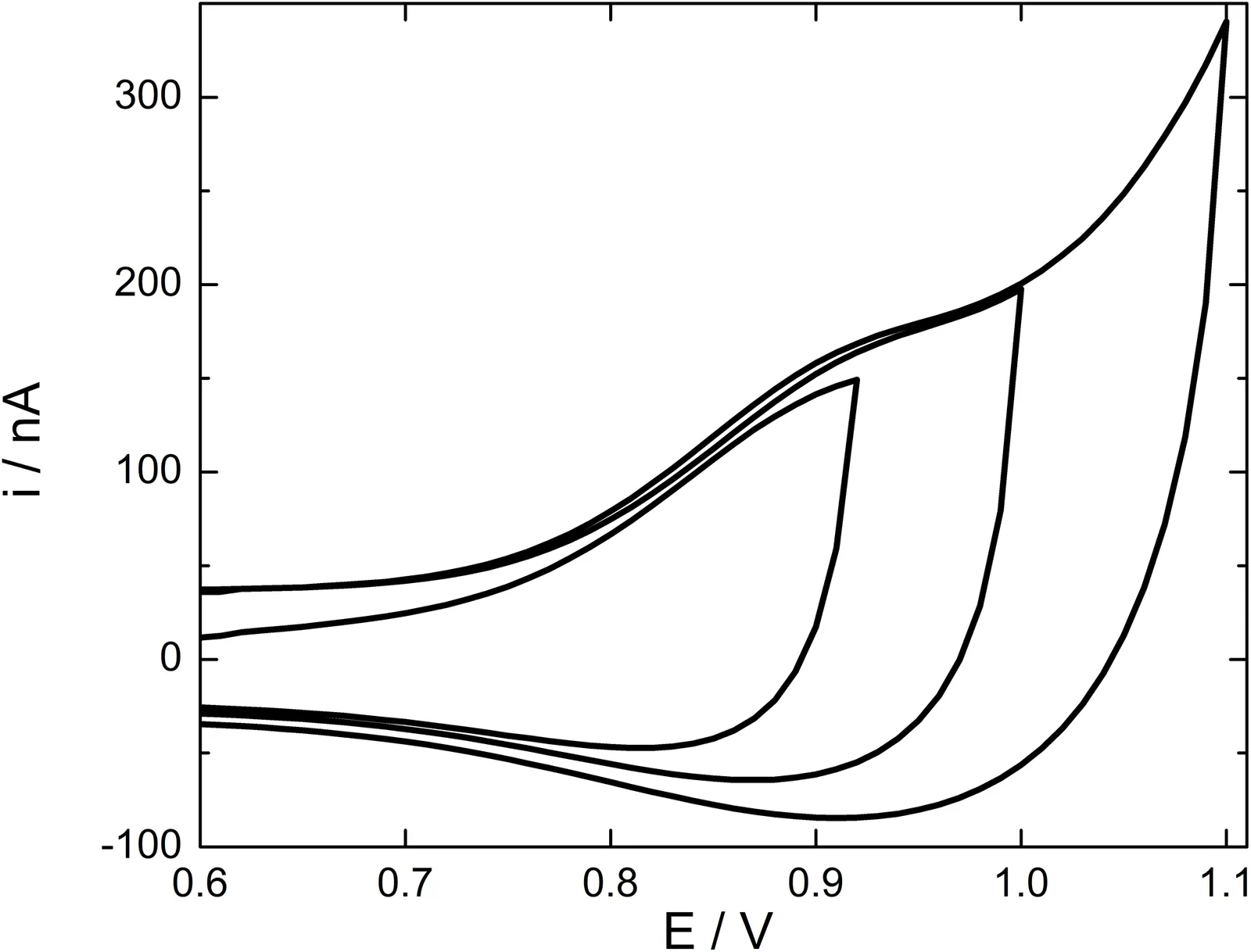
Cyclic voltammetry in LB layer. The voltage scan was reversed at three different potentials. The voltage scan rate was 50 mV s^−1^. The aqueous phase was 0.1 M NaClO_4_.

An unusual effect of a clear break in the slope of the oxidation curve occurs when voltammetric scan direction is reversed during the forward scan – the absence of reverse oxidation half-wave. This is shown in Fig. 4. The same effect was observed in a previous report.^1^ We stress that both zinc porphyrin and the tested ferrocene derivative are reversibly oxidized in solutions, however, but not as LB films. We recall the work of Frumkin^34^ a century ago. He confirmed two major findings: (i) electrostatic interactions between polarized electrodes and charged redox-active species alter reactant concentrations at the reaction plane, and (ii) diffuse charge screening and capacitive electric double layer effects alter the effective driving potential of a redox reaction. This can lead to local deviations from theoretical “bulk” conditions. The Frumkin electrostatic effect holds for the anode–cationic interaction with the tip still inside the surface layer. We consider the electrostatic repulsion as the most likely explanation for the lack of reversibility during the voltammetric back voltage scan. The electrostatic repulsion is in agreement with the most recent review.^29^

Another complication is a very small, but not negligible solubility of solvents in water (Table S1). Chloroform was used for forming the surface layer. The trace solubility in water was monitored by UV-vis spectra (Fig. S6 and Table S1).

Furthermore, Brewster angle microscopy (BAM) images, obtained using our in-house instrument (see Fig. S3) revealed that, following solvent evaporation, the porphyrin molecules formed large crystal conglomerates on the aqueous surface (with or without electrolyte), rather than a uniformly distributed film (Fig. S7), which is consistent with previous studies of porphyrins on LB.^35^ This observation further suggests that the formation of a single-crystalline polymer with large lateral dimensions is unlikely.

### 3.3. Chronoamperometry on LB film at a constant potential

**Fig. 5:**
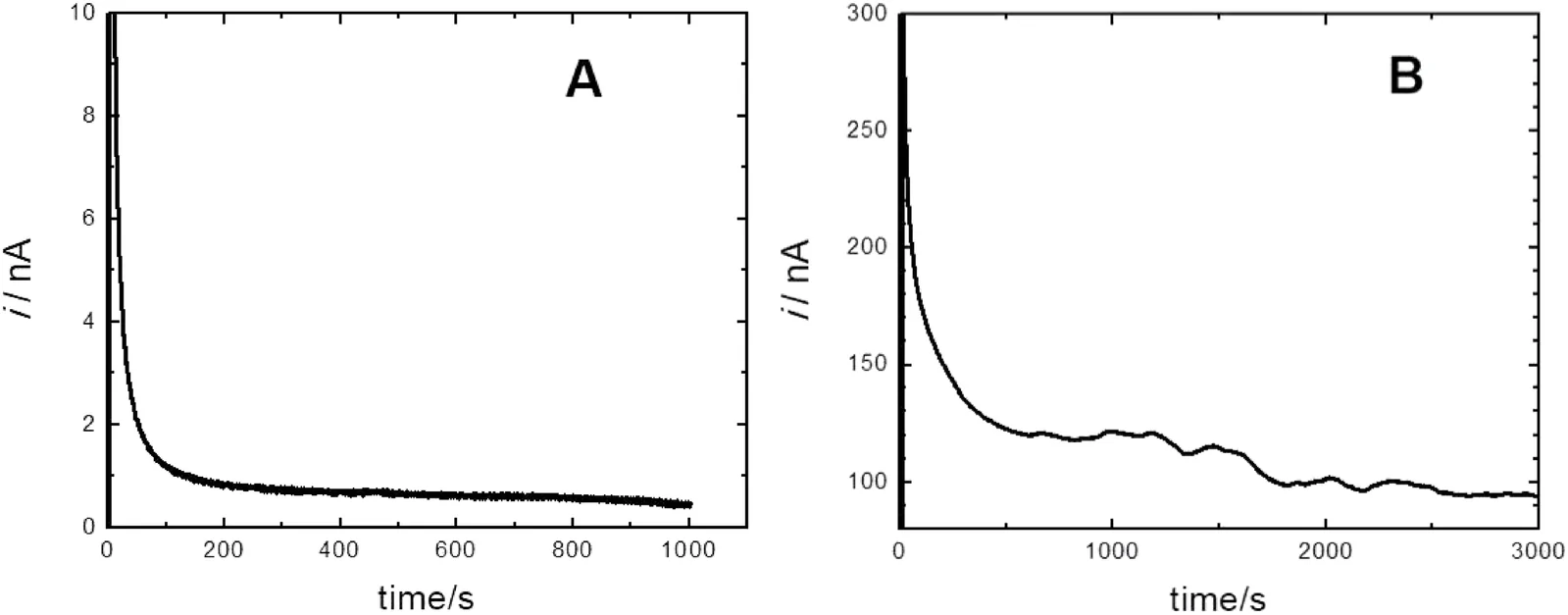
Chronoamperometry in the surface layer using STM-type tip with the surface area 0.9 µm^2^. The potential 0.67 V is the onset of a CV current (A) and the current maximum of a CV at 1.4 V is in (B). The aqueous phase was 0.1 M NaClO_4_.

Two small reduction peaks at 0.6 V in Fig. 3C suggest a possible formation of a dimer. Many similar switching of dimer formation–dimer reduction were reported.^27^ Chronoamperometry of the Zn porphyrin film at aqueous surface is shown in Fig. 5. The application of potential 0.67 V corresponds to the initial part of the oxidation current. The current–time decay smoothly approaches zero value (Fig. 5A). Increasing the applied potential to 1.4 V yields a multi electron oxidation and currents in the range of hundreds of nA. The time dependence is irregular with many fluctuations (Fig. 5B). This observation suggests a partial inactivation/activation of the electrode surface, possibly by a surface coverage by an electron transfer product (see also a CV in Fig. 2). The oxidation current decayed very slowly. During experimental time a complete concentration exhaustion of Zn porphyrin was not achieved.

Recently published^26^ chemical oxidation of a Zn porphyrin layer produced a 1.6 nm thick polymer structure given in the Chart 1. The electrochemical oxidation reported here was supposed to lead to a thin 2D layer. The potential could control the nucleation and growth producing very few supercritical nuclei of a new solid phase. If a sufficiently large critical nucleus is not formed, the cycle of nucleus growth does not repeat itself and vanishes instead.

The polymer's growth assumes that a radical-dimer (or an oligomer) will follow Scheme 1. It is assumed that a desired structure will enlarge at the periphery of a nucleus. The polymerization is certainly absent in solution oxidations (Fig. 1) because CV data show chemical reversibility. Based on Fig. 3B, it can be assumed that the potentials 1.2 V or higher produce multi-electron transfers, which promotes the nucleation of a critical nucleus of oxidized Zn porphyrin followed by the growth of a new solid phase. A repeated reaction of the parent Zn porphyrin and a radical Zn porphyrin˙^+^ formed by electron transfer (ET) at the growing periphery of a 2D structure, is proposed (Scheme 1). However, this mechanism assumes electric conductivity of the environment and/or substrate for ET from the STM-type tip electrode to the periphery of the structure and oxidation of Zn porphyrin to a radical at a remote site. The insulating behavior of the oligomer may have become an unexpected complication. A recent report^26^ proved that the Zn-porphene (Chart 1) is an insulator, unless it is doped with iodine. Simultaneous doping and radical production is impossible for present electrochemical polymerization.

The dimerization rate is the second order reaction of radical concentrations (Zn porphyrin˙^+^).^6^ At a larger STM tip (see Experimental section) and more positive potentials (>1.4 V), the reduction double peak was observed confirming the dimer formation (Fig. 3C).

### 3.4. Scanning tunneling microscopy (STM)

**Fig. 6:**
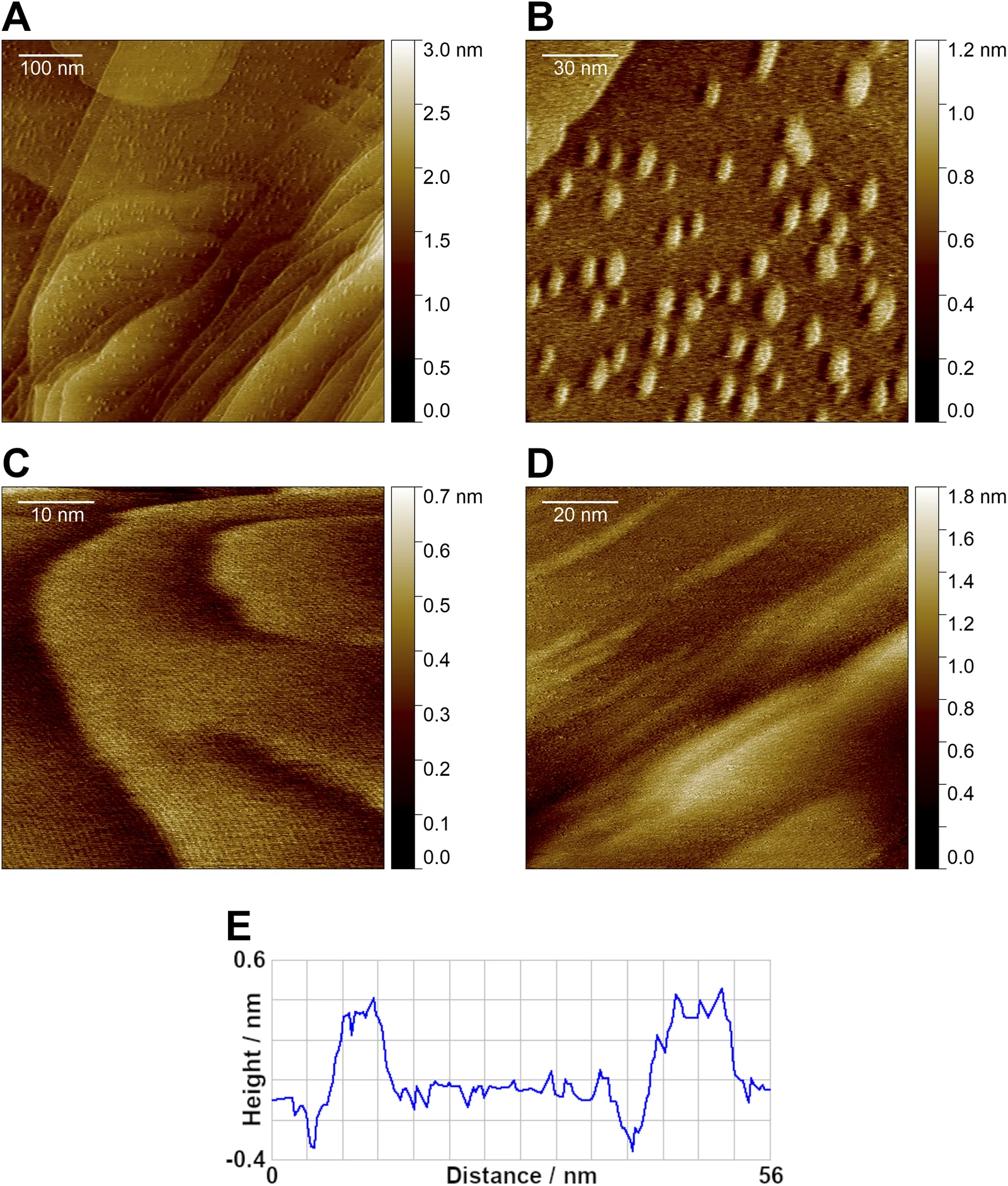
STM image of Au(111) surface deposited with LB film after 3 hours of oxidation at 1.4 V (A). Increased resolution image of Au(111) surface deposited with LB film after 3 hours of oxidation at 1.4 V (B). Au(111) surface after contact with aqueous phase but without Zn porphyrin in the system (C). Au(111) surface with Zn porphyrin drop-casted on it and evaporated, in absence of electric current (D). Examples of height profiles of formed islands from Fig. 6B (E).

**Fig. 7:**
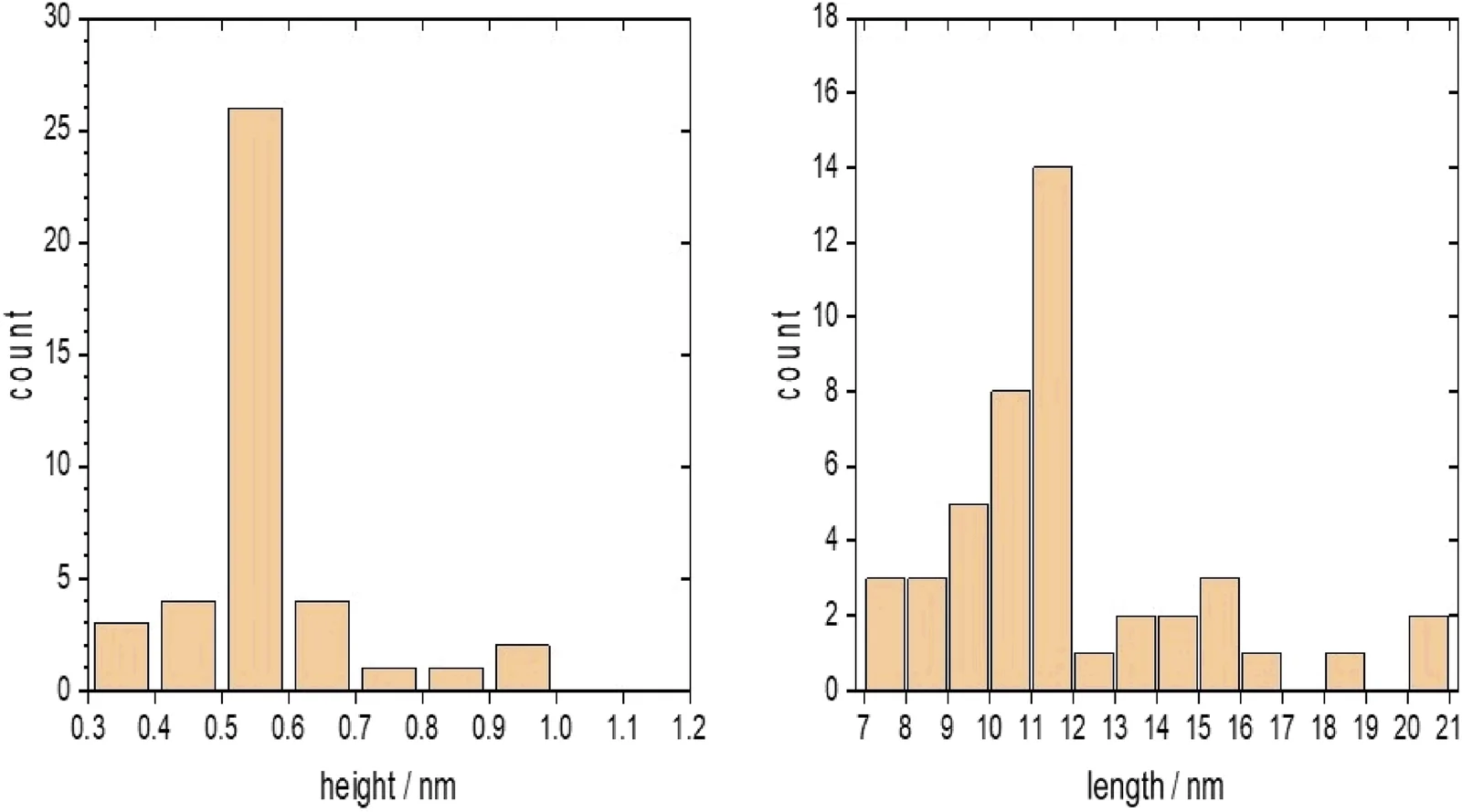
Histogram of nuclei height (left) and length of “foot print” (right) of the nuclei showed in Fig. 6A and B.

Chronoamperometry at 1.4 V was performed over 1 hour and for 3 hours. Samples for the STM analysis used Au(111) plates and were prepared by dipping in the aqueous phase and lifting through the surface layer, followed by gentle washing with ultrapure water and drying. The product formed by electrochemical oxidation in LB changed the originally smooth Au substrate surface to a structure with many small islands of a nanometer size (Fig. 6A–E, 7 and S8). These island formations were not observed when the Au substrate was in contact exclusively with the 0.1 M NaClO_4_ aqueous phase (Fig. 6C), nor when a thin film of Zn porphyrin is drop-casted on the Au surface and in the absence of electric current. These data showed that the island formation observed in Fig. 6A and B are the exclusive result of passing an electric current through the Zn-porphyrin film adsorbed on the 0.1 M NaClO_4_ aqueous phase. If one of the components of the system (Zn porphyrin or electric current) was missing, the island formation was not observed. This growth model yields isolated islands of a new phase. The formation of phase transition (without oxidation) was observed in long alkyl substituted of tetraphenyl porphyrins.^35^

The average height of the most abundant nuclei is 0.525 ± 0.041 nm. The average length of islands on the Au surface gives the highest count 11.56 ± 0.64 nm (Fig. 7). The estimated height is rather uniform. The length distribution shows a considerable contribution of smaller and larger agglomerates, which would indicate the progressive type of nucleation. Based on these observations, chances to achieve considerable coverage of the surface layer by a new structure are virtually null in a reasonable time span. The growth involves the Volmer–Weber model called “island growth”. This occurs when the molecules or atoms in the deposit are more strongly bound to each other than to the substrate (in this case the surface of the aqueous phase). This growth model yields isolated islands of a new phase, as can be seen in Fig. 6B.

### 3.5. X-ray photoelectron spectroscopy (XPS)

We have observed that type of the electrolyte in the aqueous phase plays a role in the island formation during the oxidation process. Two salts were tested (Bu_4_PF_6_ and NaClO_4_) and both yielded similar island-like features as in Fig. 6B. Islands were formed in abundant amount when the LB aqueous phase contained NaClO_4_.

**Fig. 8:**
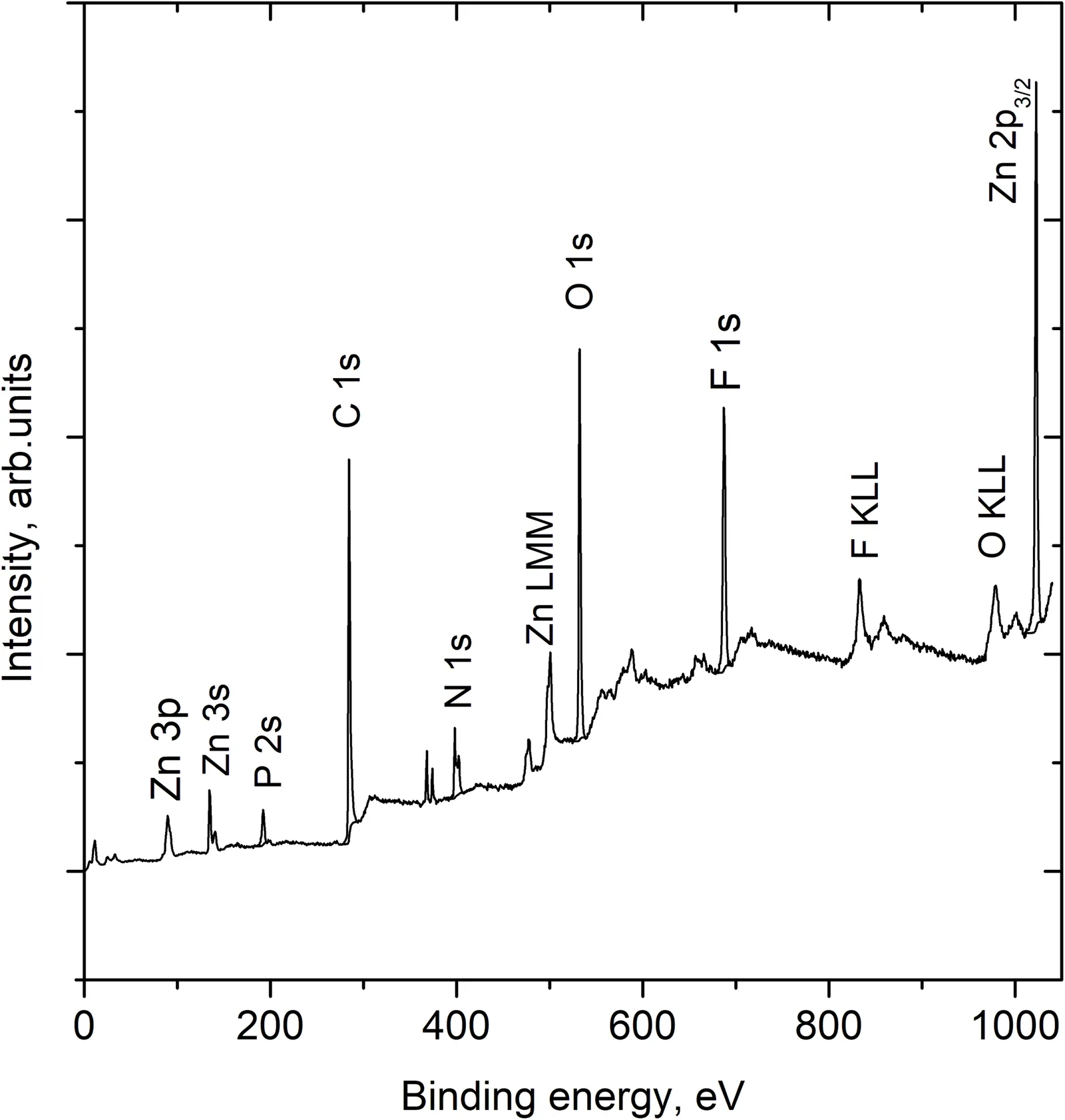
XPS spectra of oxidized zinc porphyrin deposit using 1,2-dichloroethane as a solvent and 0.1 M Bu_4_PF_6_ as an electrolyte.

**Fig. 9:**
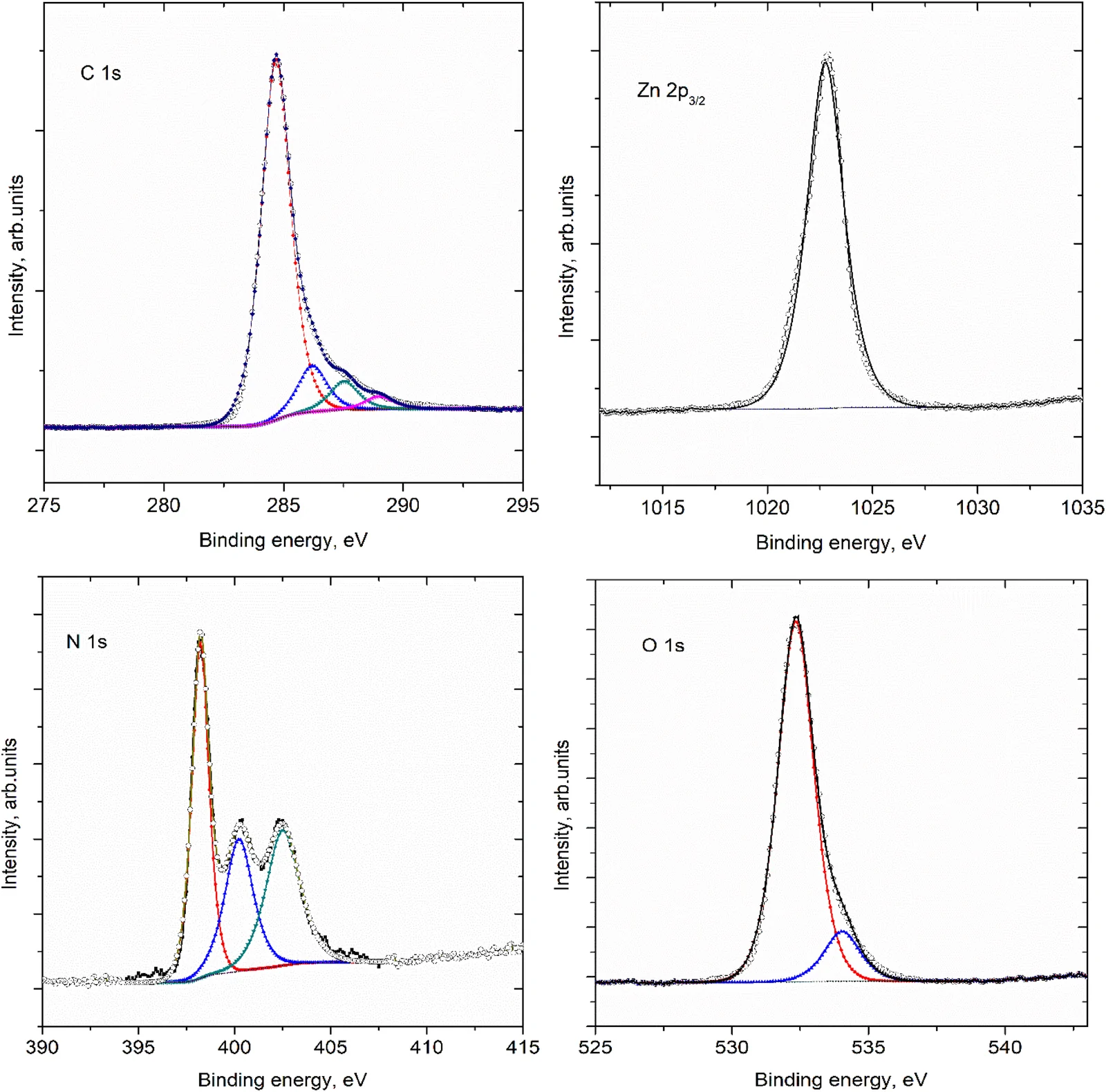
Deconvoluted selected XPS spectral lines.

To characterize better the formed oxidation product, the XPS spectra of deposited product of a bulk solution oxidation of zinc porphyrin in acetonitrile–water mixture (8 : 2) and NaClO_4_ was measured and yielded 1% atomic of Zn deposited, and two typical nitrogen maxima. Deposition of oxidized Zn porphyrin on Au(111) plate from 1,2-dichloroethane and Bu_4_PF_6_ solution were characterized by XPS spectroscopy (Fig. 8 and 9). The dark film deposit produced at +1.65 V yielded 22% of Zn, and additional third N peak (402.5 eV) corresponding to butyl cation (Fig. S9). Phosphorus and fluorine peaks were also detected originating from hexafluorophosphate anion (PF_6_^−^). Energy at 402.5 eV corresponds to tetrabutylammonium cation deposited from the supporting electrolyte (see the presence of phosphorus and fluorine atoms). Similar results were obtained at the oxidation potential at 1.35 V. This confirms the precipitation of an insoluble salt of Zn^2+^ and PF_6_^−^. Our interpretation in terms of formation of insoluble precipitates between oxidized Zn^2+^ and a suitable anion is in agreement with very recent review^29^ listing the electrostatic repulsion anode ↔ cation (in the adsorbed layer) and cation–anion (in the water phase).

Other XPS peaks are in Fig. S9. Concentrations of elements at two oxidation potentials: 1st wave and 2nd wave (in atomic%) are given in Table S2.

### 3.6. Raman spectroscopy of electrolytically prepared Zn porphyrin-based deposit

Raman spectroscopy of electrochemically oxidized Zn-porphyrin was obtained from a solution in 1,2-dichloroethane as a dark deposit on Au(111) plate. The observed Raman peaks at 1460 cm^−1^, 1499 cm^−1^, 1555 cm^−1^, and 1602 cm^−1^ produce a broad peak at around 1600 cm^−1^ in the Raman spectra.^36^ This band is a result of principally C–C bond stretching *ν*(C–C) within rings and rocking of their H, *ρ*(CH) NH pyrrole deformation.

**Fig. 10:**
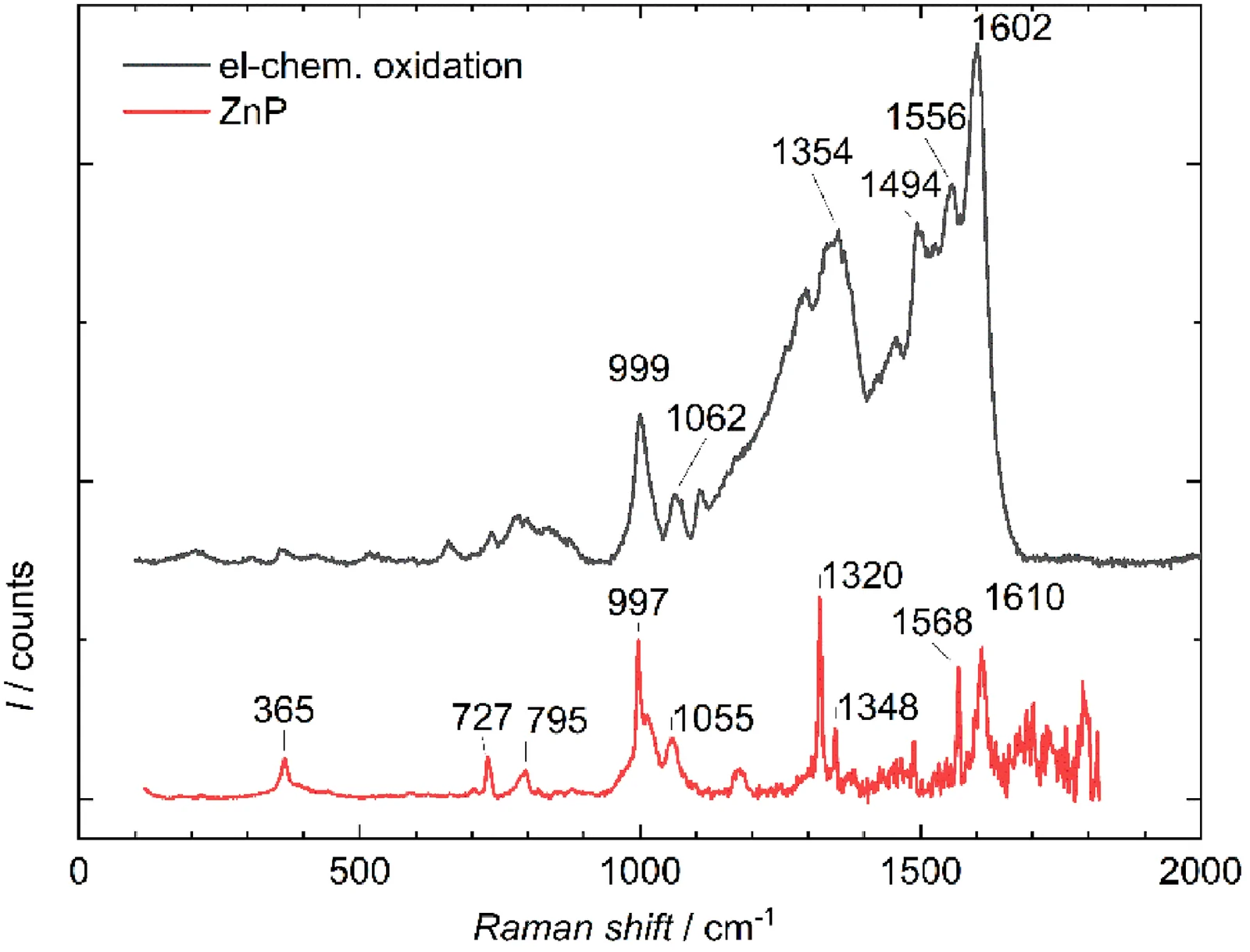
Raman spectrum of Zn-porphyrin powder (lower red curve) and electrochemically oxidized black deposit of Zn-porphyrin (upper black curve).

There are two Raman peaks that are affected by deprotonation corresponding to NH stretch at 1297 cm^−1^ and 3350 cm^−1^. The band at 1002 cm^−1^ is caused by expansion of the pyrroles along N(H)⋯N(H) direction likewise macrocycle breathing (or breathing of pyrroles in the same phase) as assigned by Rich and McHale.^37^ Zn porphyrin dark deposit measured at different spots of Au substrate (Fig. S10) are identical (Fig. 10).

Experimental data suggests the following interpretation:

(i) The reversible reduction of a radical (the first CV wave) is not observed for zinc porphyrin in a LB layer. Electrostatic repulsion of cations from the positive charge of the electrode tip (Frumkin effect) is responsible for spreading small nuclei away from the electrode tip over the interfacial layer, and thus for irreversibility of the oxidation. It is exactly the same behavior as observed for the oxidation of a ferrocene derivative layer in a LB trough.^1^

(ii) Larger domains do not form because the mechanism should involve electron–hole transfer to the remote periphery. However, only the small islands of polymer obtained from Zn porphyrin thus prevent long distance electron transfer to the periphery site. This is a result of the Frumkin electrostatic repulsion.

(iii) Topography profile determined by STM indicates that the height (0.52 nm) fits the thickness of two layers in an insulated island of a nucleus. The average length of the nuclei (islands) is 11.6 nm which might correspond up to 14 fused porphyrin rings, based on the published values.^14^

(iv) Volmer–Weber mechanism of growth suggests a stronger interaction among the oxidized product compared to the interaction of the new phase with the electrode. This contributes to spreading the small nuclei over the surface.

(v) XPS spectroscopy of a dark deposit obtained through electrochemical oxidation on Au(111) plate at 1.65 V proved the presence of carbon, nitrogen, and zinc besides other elements. Consistent results were obtained at other oxidation potentials and liquid media. Similar behavior yields the oxidation at the potential of the first oxidation potential (1.35 V).

(vi) Raman spectroscopy of electrochemically oxidized Zn-porphyrin deposited on Au(111) from solution bulk oxidation agrees to Raman spectroscopy data published previously.^36^

## 4. Conclusions

The first application of the electrochemical Langmuir–Blodgett trough resulted in an adequate functioning. Electrochemical polymerization of zinc porphyrin yielded considerably thinner fragments, grown by the Volmer–Weber mechanism. The electrostatic repulsion between cations and the positive potential of the electrode resulted in the dispersion of islands of a new phase over the surface of adsorbed layer. Isolated islands were subsequently observed after their transfer to the Au(111) substrate. The height of the most abundant nuclei is 0.52 ± 0.04 nm. The size histogram of the obtained islands on the surface indicates that the dominant island length is ∼11.56 nm. Electrochemical oxidation proves to be an alternative method for the synthesis of porphene, without the need for a chemical oxidant. Electrochemical oxidation products were characterized by STM topography, XPS, and Raman spectroscopy.

## Author contributions

K. Majerová Varga: investigation, F. Vavrek: investigation, V. Urbanová: investigation, J. Plutnar: investigation, Z. Bastl: investigation, M. Pazderková: investigation, L. Ludvíková: investigation and editing, P. I. Dron: investigation, writing, review and editing, M. Hromadová: writing, review and editing, L. Pospíšil: methodology, electrochemistry and writing.

## Conflicts of interest

The authors declare that they have no known competing financial interests or personal relationships that could have appeared to influence the work reported in this paper.

## Supplementary Material

RA-016-D6RA05196K-s001

## Data Availability

Data for this article, including cyclic voltammetry and Raman spectra are available at Zenodo repository at https://doi.org/10.5281/zenodo.20590253. Supplementary information (SI): electrochemistry, Raman spectra. See DOI: https://doi.org/10.1039/d6ra05196k.
