# Supplementary material for: Oxidative polymerization of zinc porphyrin using the electrochemical Langmuir–Blodgett trough
Source: RSC Adv. 2026 Aug 18;16(41):48823–33. doi: 10.1039/d6ra05196k (PMC13505409; doi:10.1039/d6ra05196k)
Supplement: Supplementary file 1 [file RA-016-D6RA05196K-s001.pdf]

Supplementary Information (SI) for RSC Advances.
This journal is © The Royal Society of Chemistry 2026

Supporting Information

Oxidative Polymerization of Zinc Porphyrin on Electrochemical Langmuir-Blodgett Trough.

K. Majerová Varga,[a] F. Vavrek,[b] V. Urbanová,[a] J. Plutnar,[a] Z. Bastl,[b] M. Pazderková[a], L. Ludvíková,[a] P. I. Dron,[a,c]* M. Hromadová,[b] L. Pospíšil,[a,b]*

[a] *Institute of Organic Chemistry and Biochemistry Czech Academy of Sciences, Flemingovo nám. 542/2, 160 00 Prague, Czech Republic;*
[b] *J. Heyrovský Institute of Physical Chemistry, Czech Academy of Sciences, Dolejškova 2155/3, 182 23 Prague 8, Czech Republic;*
[c] *Department of Chemistry, Middle East Technical University, Ankara, 06800, Turkey*

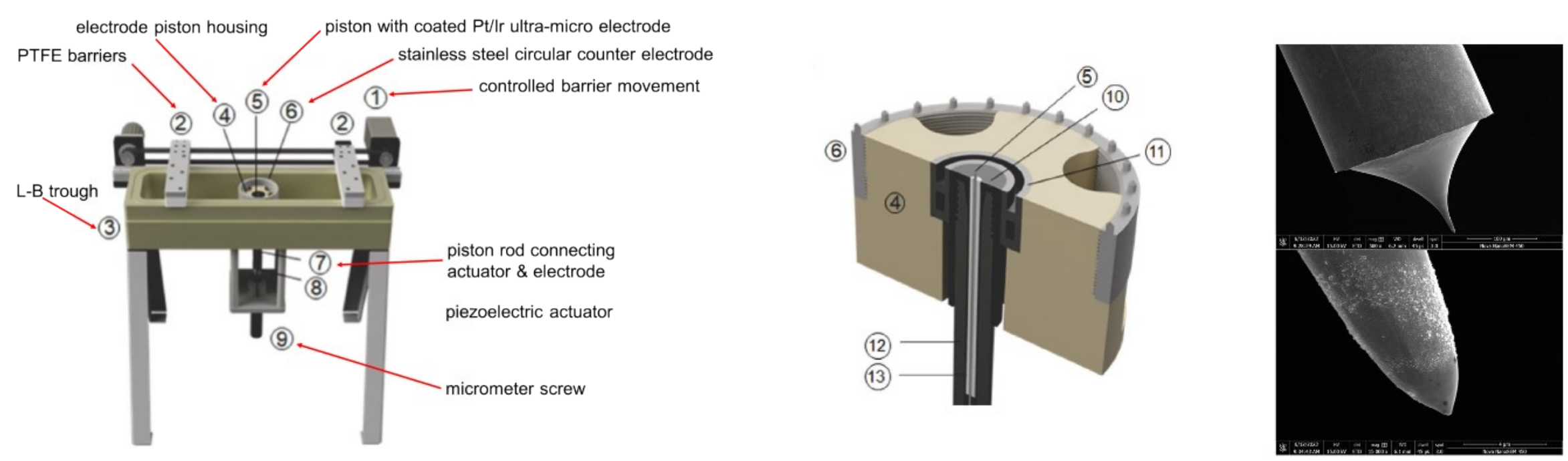

Figure S1. The electrochemical LB trough (left), electrodes (middle): circular cathode (6) and upward movable piston with UME anode (5), TEM image of UME anode (right). For more details, see our recent publication: Urbanová, V.; Bartl, T.; Vavrek, F.; Pačes, O.; Pospíšil, L.; Michl, J. Local cyclic voltammetry of a Langmuir-Blodgett film on water. *Langmuir* **2024,** *40,* 2562–2566.

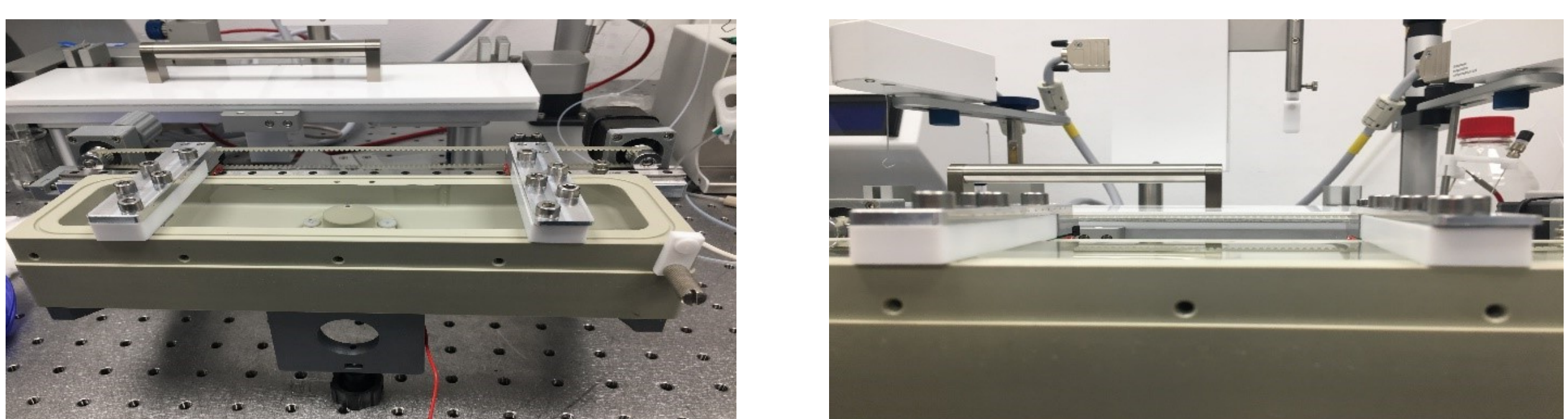

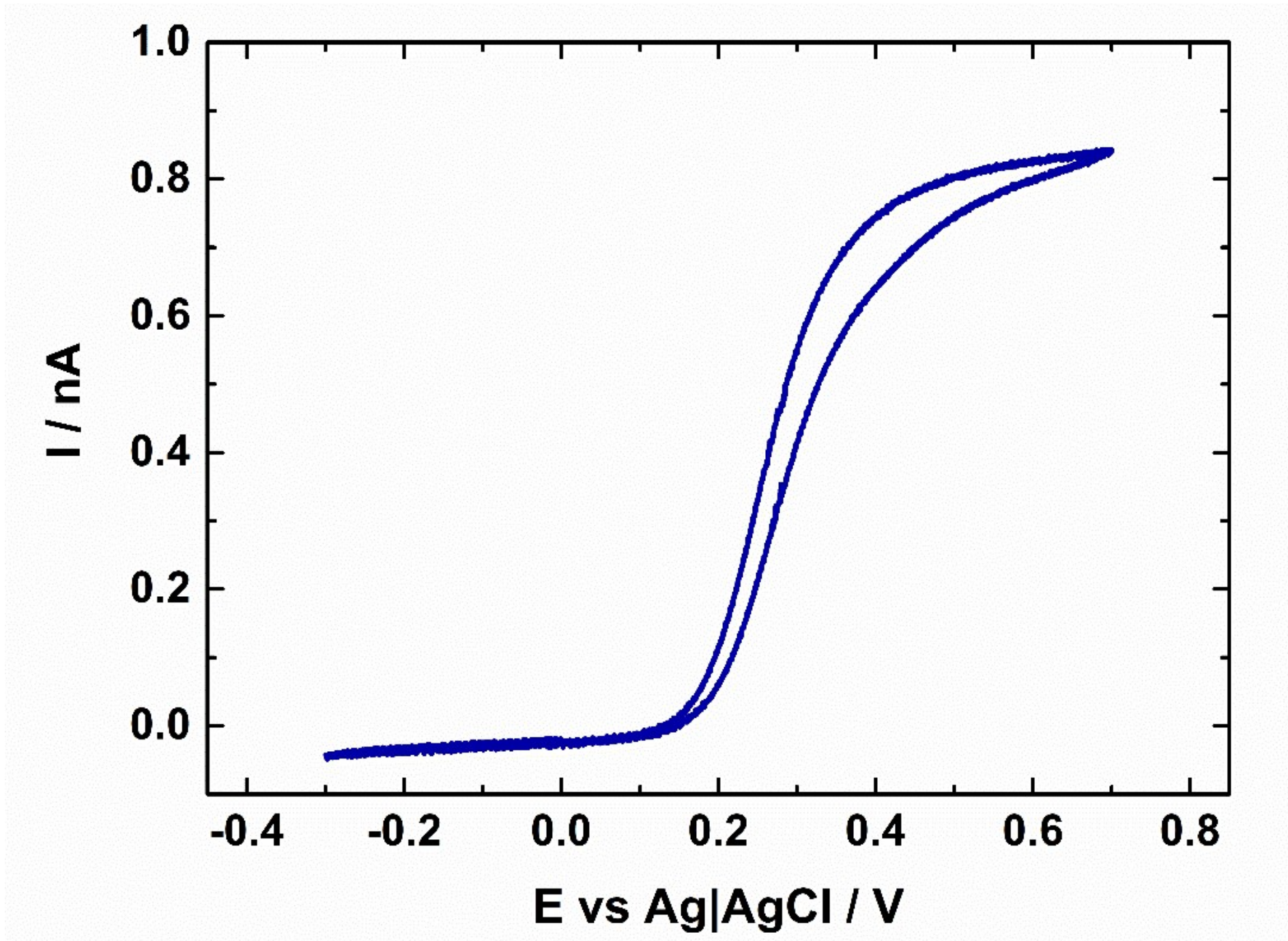

Figure S2. Representative cyclic voltammogram of 1 mM potassium ferrocyanide in aqueous 0.1 M Na_2SO_4 using STM-type UME of the area 28.6 μm^2.

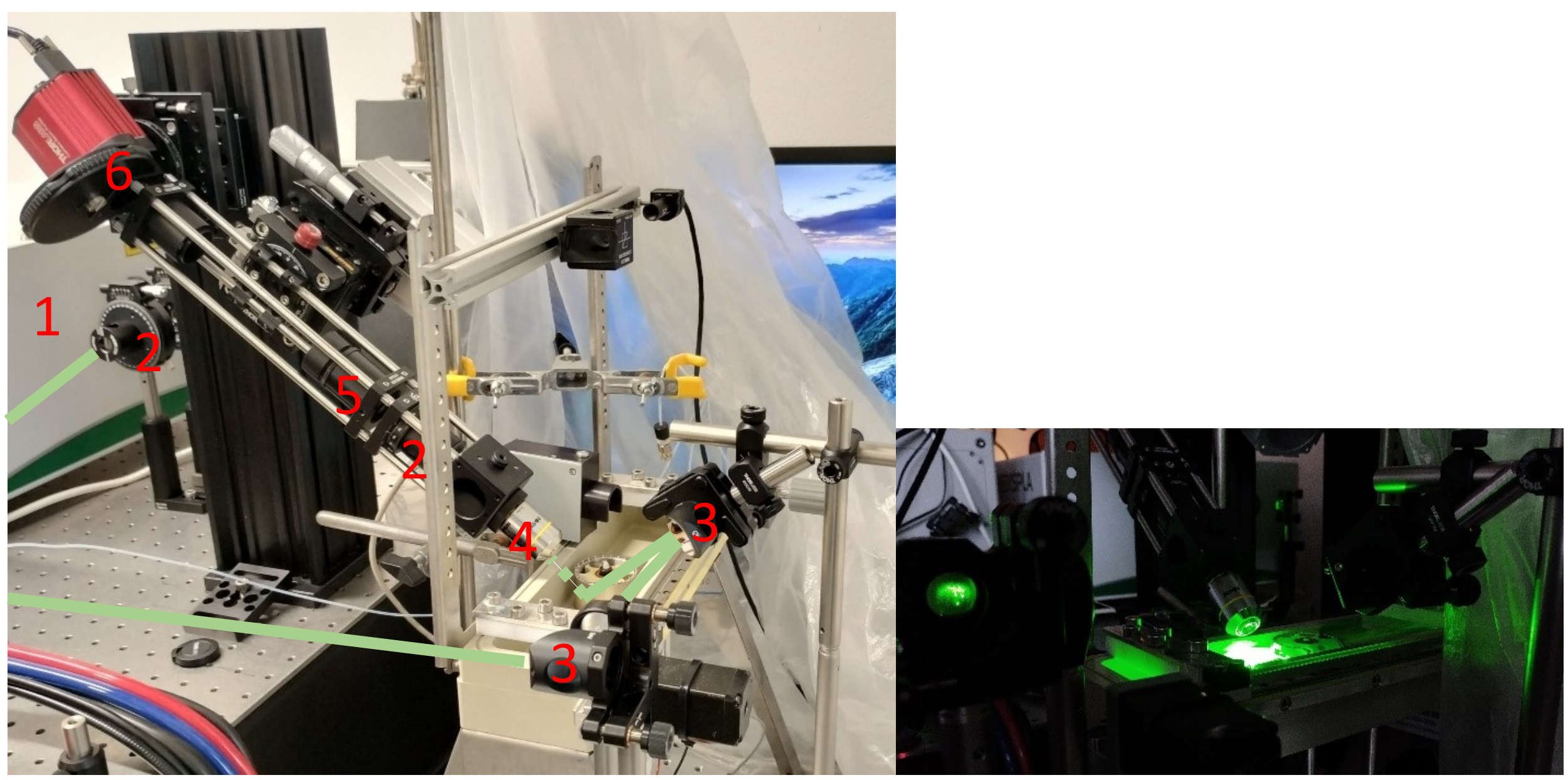

Figure S3. In-house built Brewster angle microscope (BAM) with depicted laser optical path (green) consisting of: 1 – ns-pulsed laser (Ekspla NT242) tuned to 532 nm, 2 – two Glan-laser calcite polarizers, 3 – silver mirrors, 4 – OLYMPUS objective 10x, 5 – achromatic tube lens (175 mm FL), 6 – ThorLabsCMOS camera (2448x2048 Pixels).

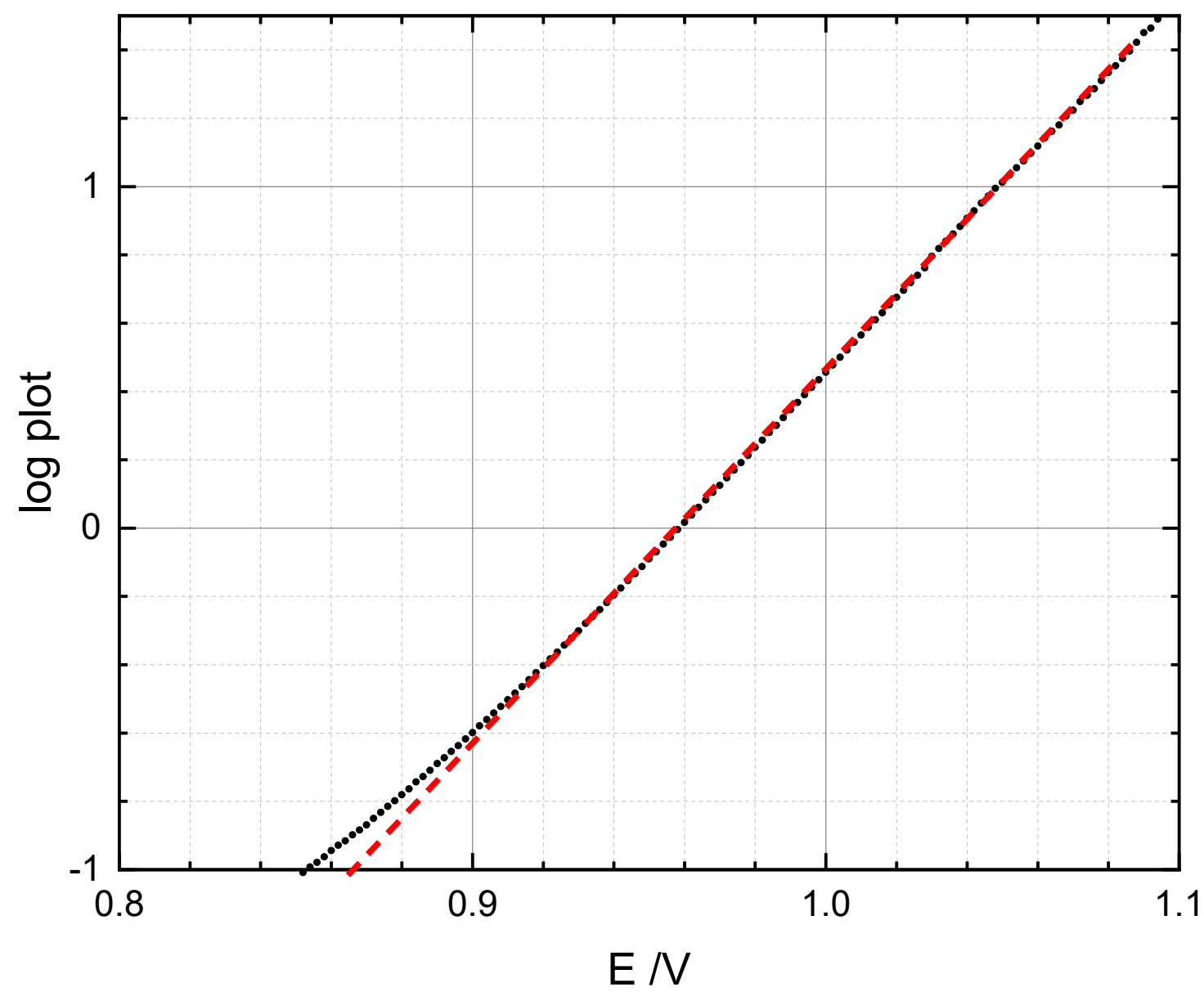

Figure S4. Log-plot analysis of Zn porphyrin using UME.

Cyclic voltammetry of ZnP using freshly polished Pt UME of diameter 10 μm in dichloroethane without an electrolyte is given in Figure 1 of the main text. The log-plot analysis of the wave shape is the dependence of log $(i/(i_{lim} - i))$ vs E. It yields a straight line (red) over two current decades, slope of which give the value of the charge transfer coefficient α = 0.5. The scan rate was 2 mV/s.

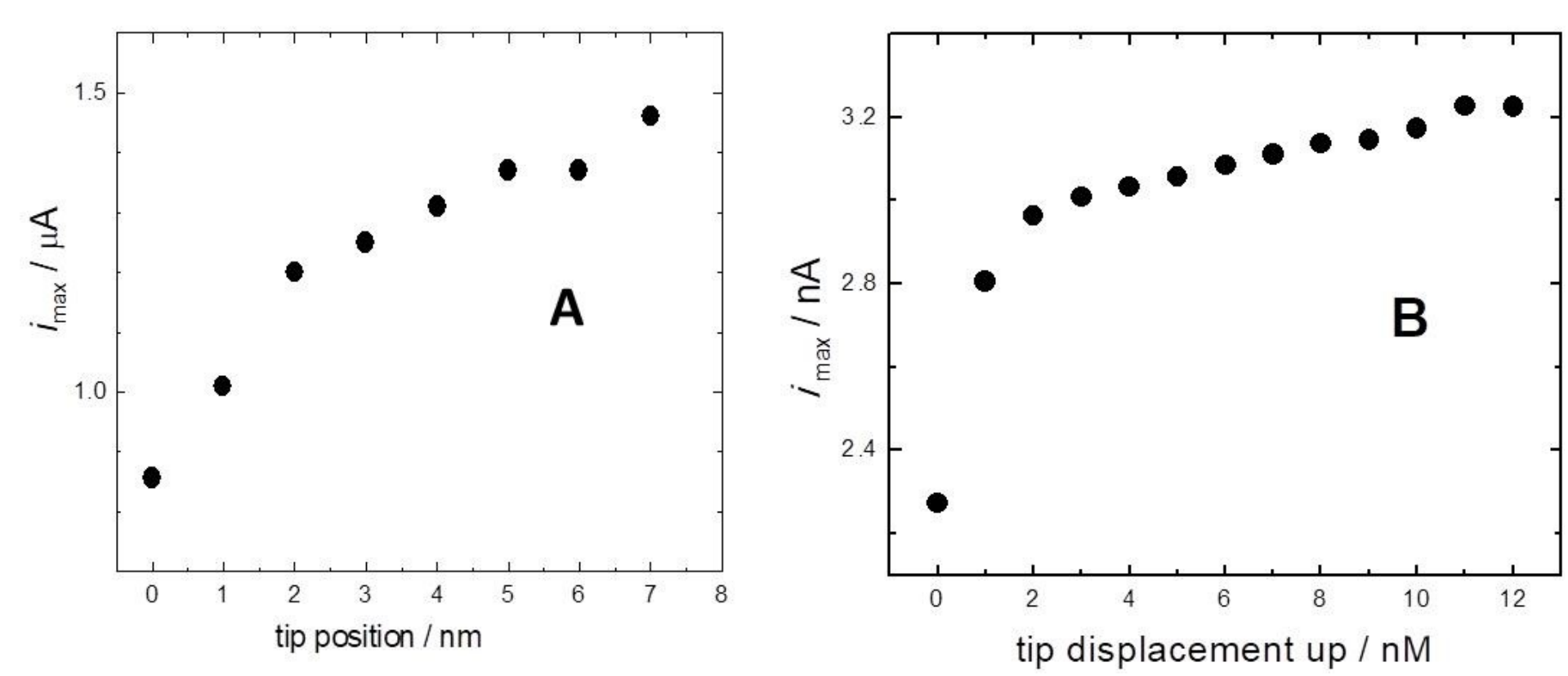

Figure S5 Maximum peak currents during the gradual approach of the tip towards the surface layer. Data shown for experiment employing UME tip with the area 4.5 μm^2 (A) and 0.9 μm^2 (B).

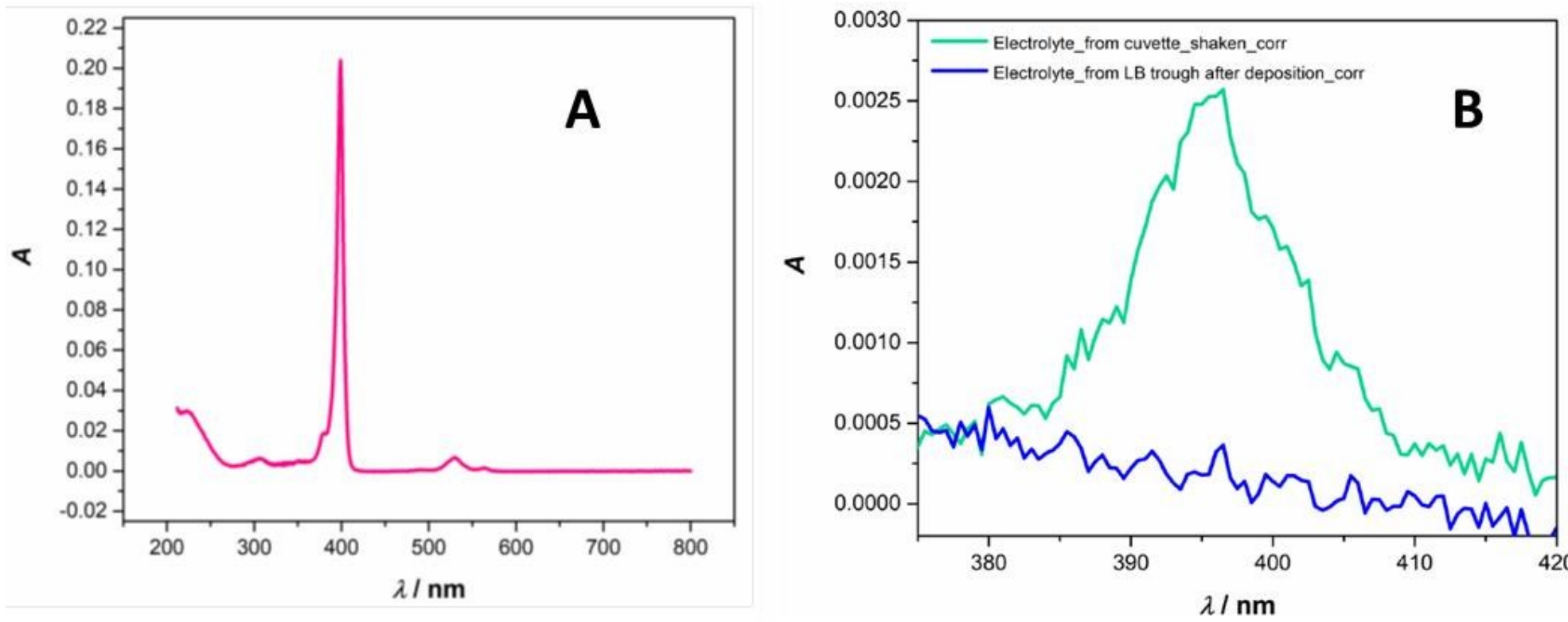

Figure S6 Solubility of solvents in water. UV-vis spectrum of Zn porphyrin in tetrahydrofuran (A) and in a cuvette with water covered by a surface layer of a solution of Zn porphyrin in chloroform (B).

Table S1 Solubility of solvents in water and solvent densities

Solvent	Solubility in water	Density
acetonitrile	miscible	0.783
benzene	0.18 %	0.88
chloroform	0.815%	1.48
1,2-dichloroethane	0.87 %	1.25

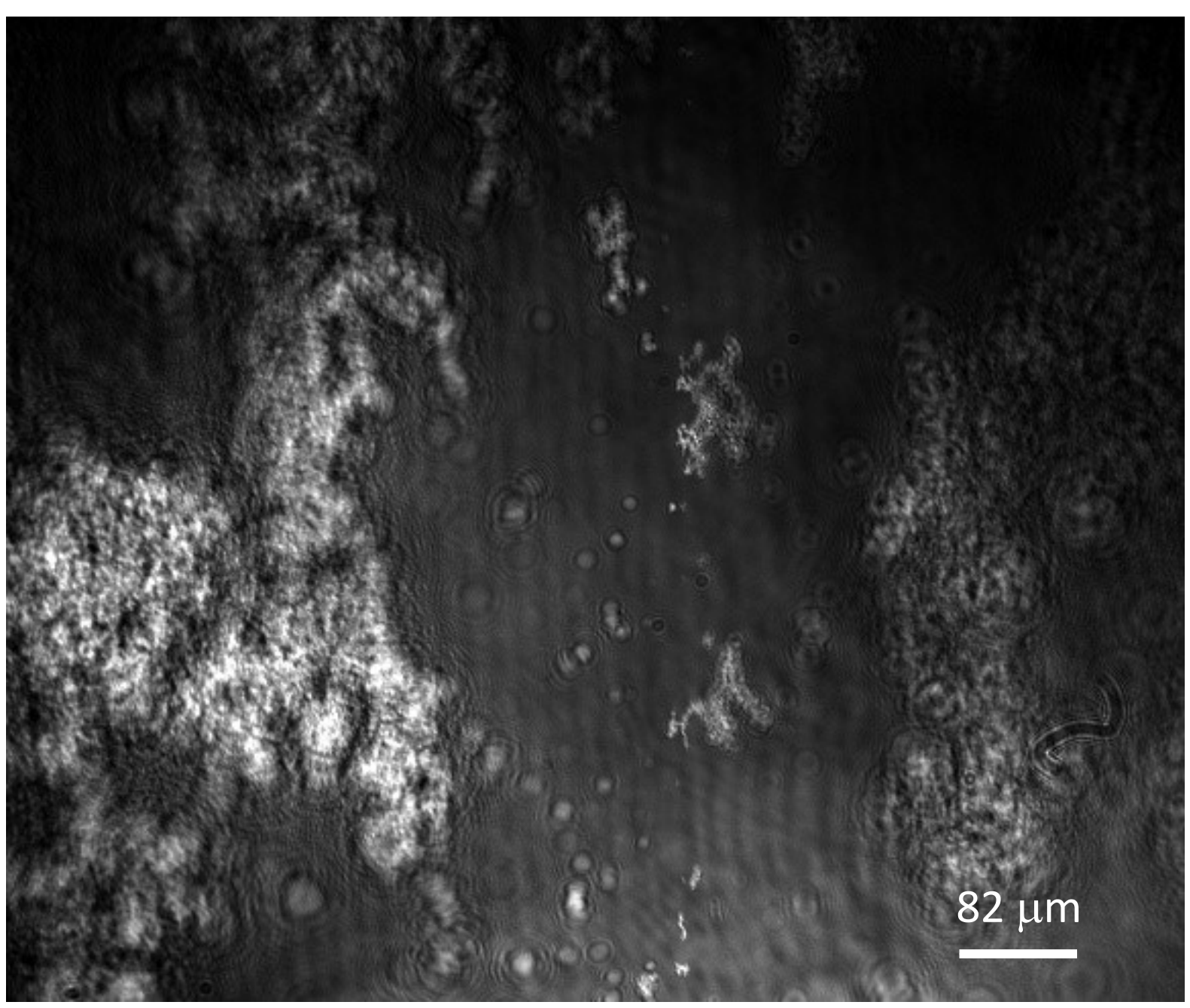

Figure S7 BAM image of Zn porphyrin (crystals in white on the left) spread on the top of aqueous surface.

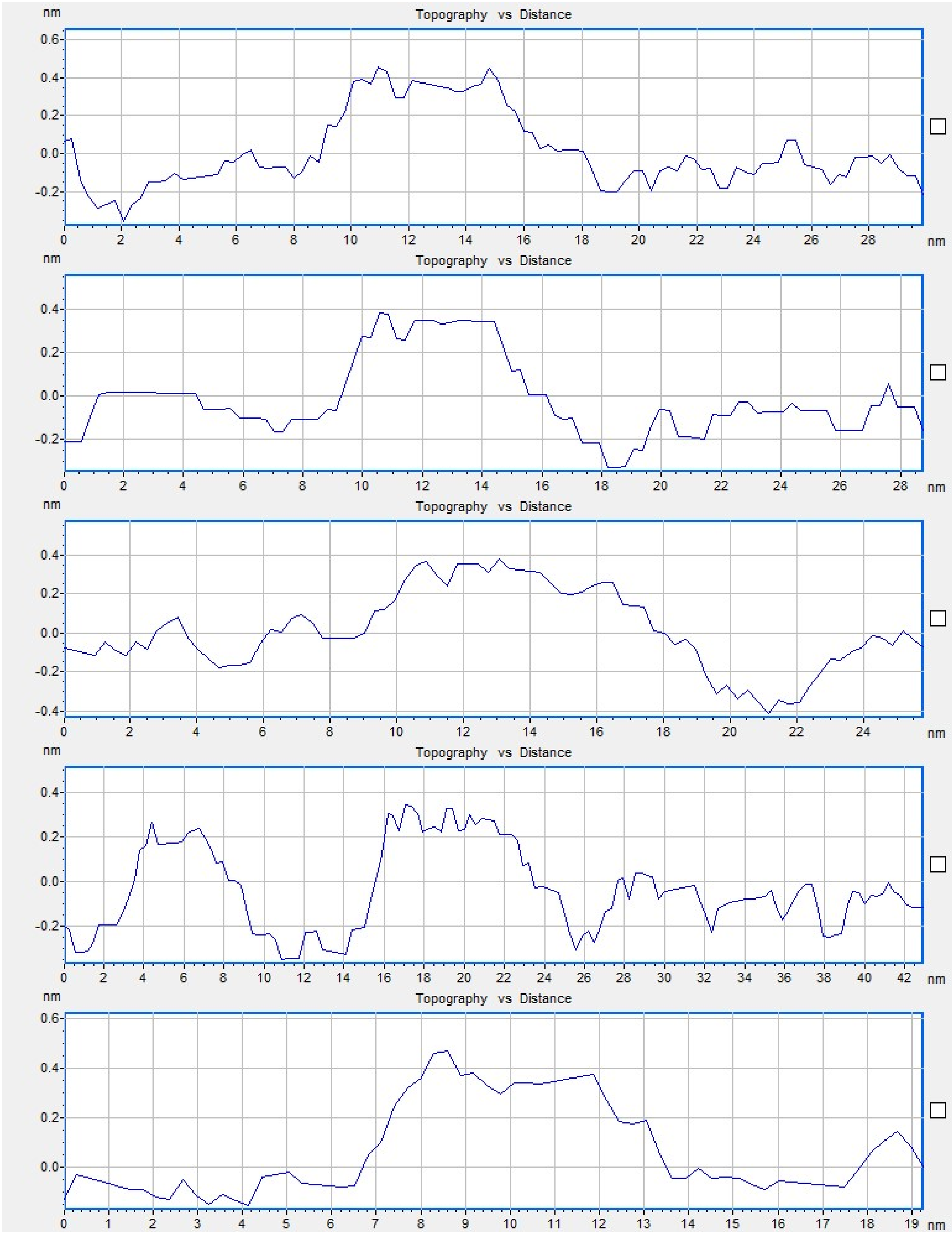

Figure S9 Additional examples of STM topographic cross-sections.

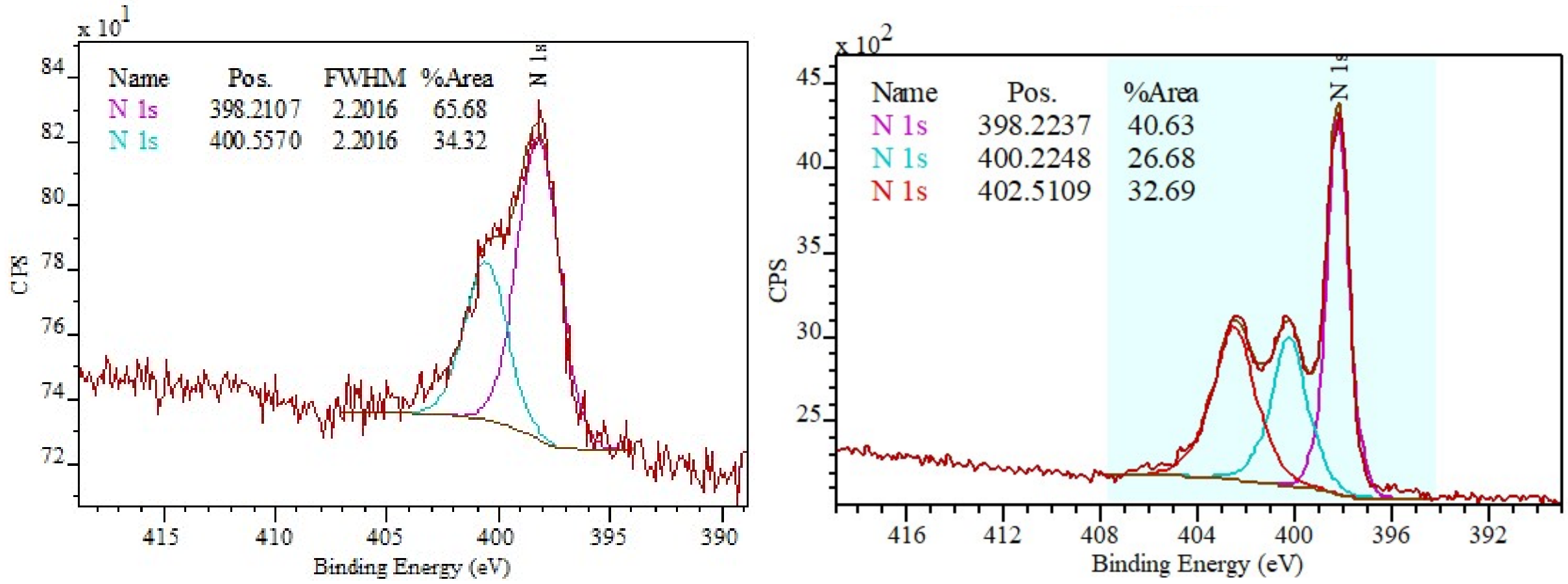

Figure S10. Binding energy of nitrogen in oxidized Zn porphyrin. Electrochemical oxidation performed in a mixture of acetonitrile:water (8:2) with $NaClO_4$ supporting electrolyte (left spectrum) and in 1,2-dichloroethane with Bu_4PF_6 supporting electrolyte (right spectrum).

Table S2

Concentrations of elements at two oxidation potentials: 1st wave & 2nd wave (in atomic %)

Element	C	Zn	N	O	F	P
Potential						
1.35 V	39.0	8.7	7.2	24.6	14.7	5.8
1.65 V	47.8	7.3	7.9	19.5	13.0	4.4

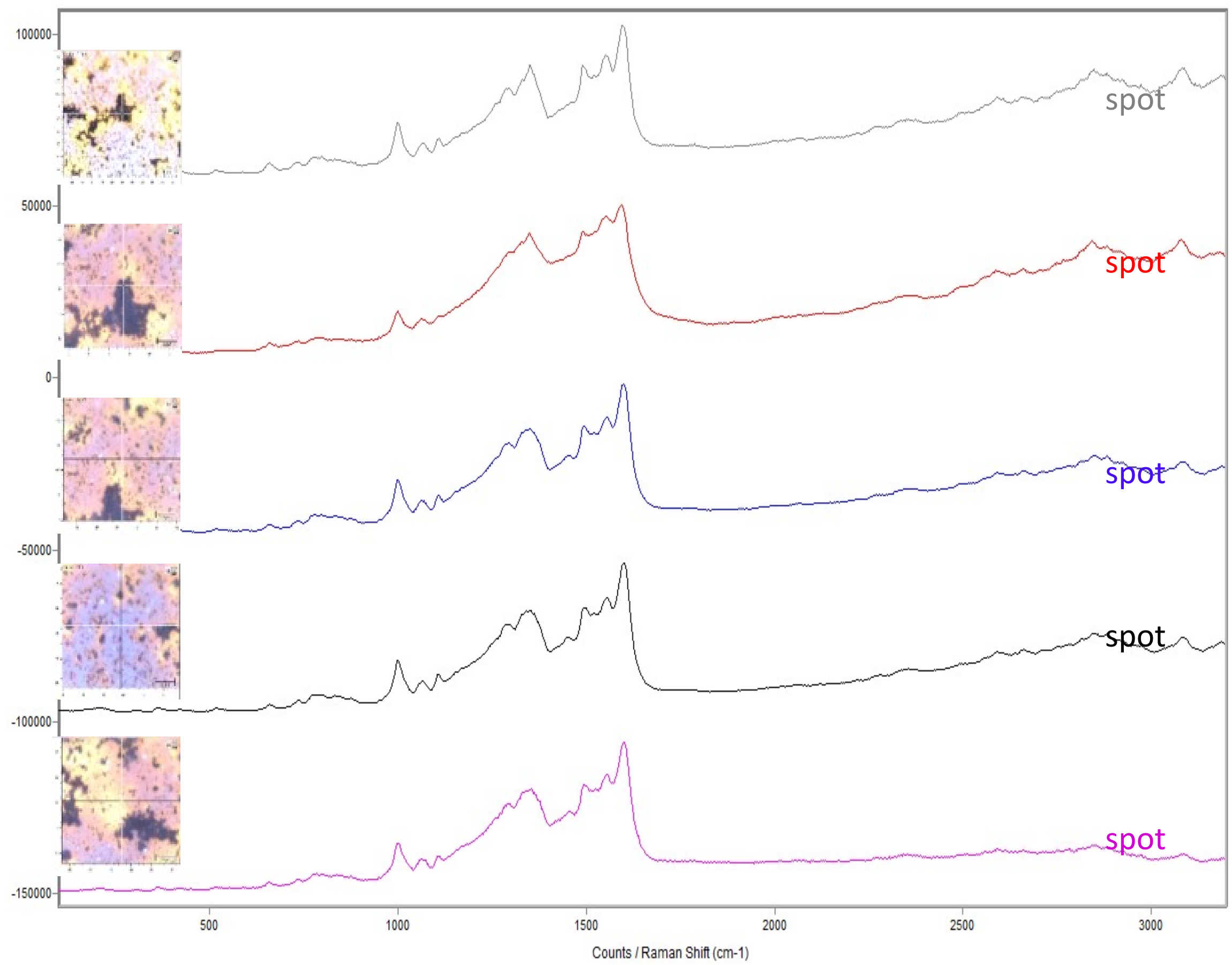

Figure S11. Raman spectra taken at different spots on the Au(111) plate.

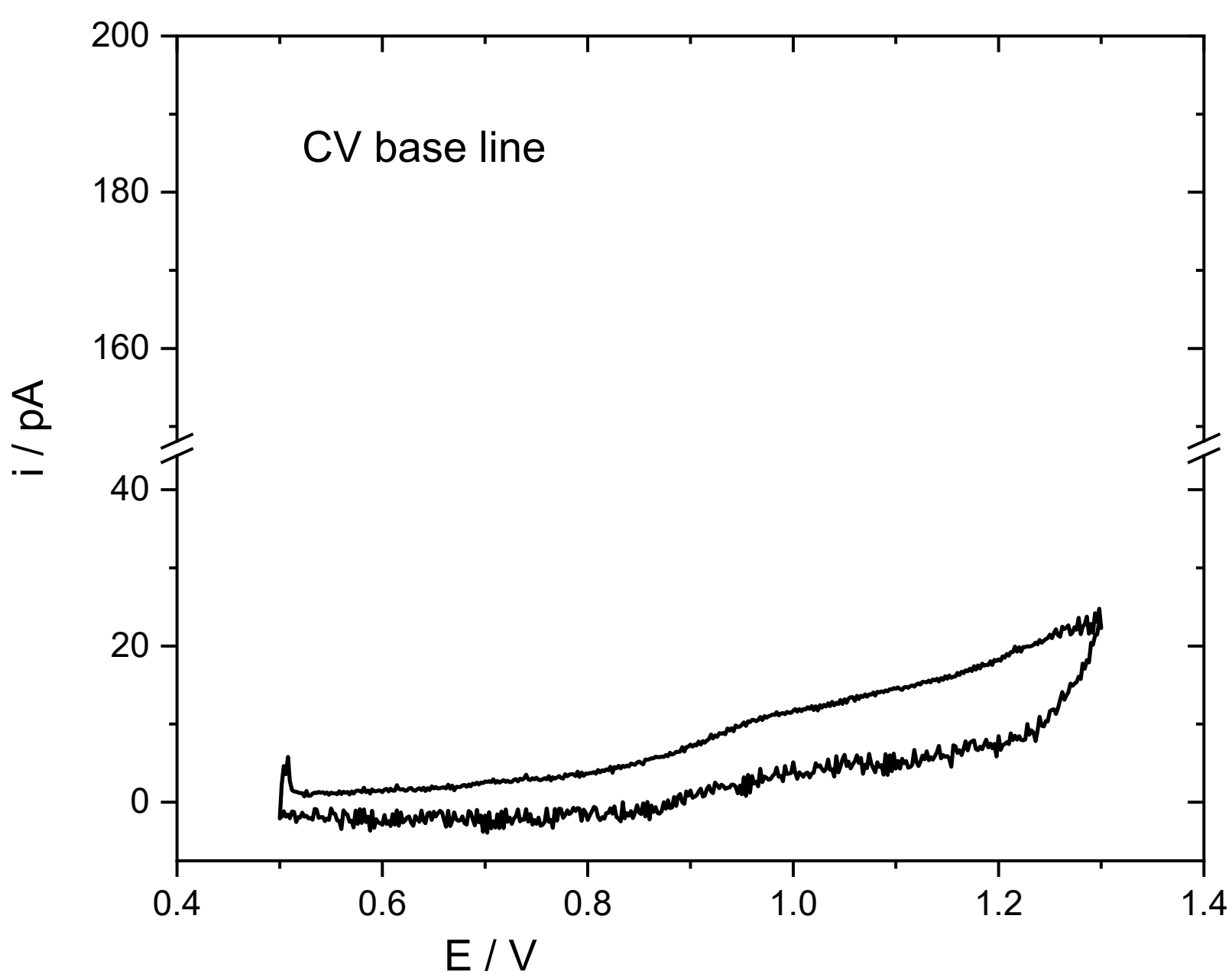

Figure S12 Cyclic voltammetry base line using 10 μM diameter UME. The voltage scan rate was 10 mV/s. The solvent was 1,2-dichloroethane.
